# Novel Intersubunit Interaction Critical for HIV-1 Core Assembly Defines a Potentially Targetable Inhibitor Binding Pocket

**DOI:** 10.1128/mBio.02858-18

**Published:** 2019-03-12

**Authors:** Pierrick Craveur, Anna T. Gres, Karen A. Kirby, Dandan Liu, John A. Hammond, Yisong Deng, Stefano Forli, David S. Goodsell, James R. Williamson, Stefan G. Sarafianos, Arthur J. Olson

**Affiliations:** a Department of Integrative Structural and Computational Biology, The Scripps Research Institute, La Jolla, California, USA; b Synsight, Évry, France; c Christopher S. Bond Life Sciences Center, University of Missouri, Columbia, Missouri, USA; d Department of Chemistry, University of Missouri, Columbia, Missouri, USA; e Division of Chemistry and Chemical Engineering, California Institute of Technology, Pasadena, California, USA; f Department of Molecular Microbiology & Immunology, University of Missouri School of Medicine, Columbia, Missouri, USA; g Laboratory of Biochemical Pharmacology, Department of Pediatrics, Emory University School of Medicine, Atlanta, Georgia, USA; h Center for Integrative Proteomics Research, Rutgers State University, Piscataway, New Jersey, USA; i Department of Biochemistry, University of Missouri, Columbia, Missouri, USA; University of Pittsburgh School of Medicine; University of Delaware; University of Chicago

**Keywords:** X-ray crystallography, capsid, capsid assembly, computer modeling, human immunodeficiency virus

## Abstract

HIV-1 capsid protein (CA) plays critical roles in both early and late stages of the viral replication cycle. Mutagenesis and structural experiments have revealed that capsid core stability significantly affects uncoating and initiation of reverse transcription in host cells. This has led to efforts in developing antivirals targeting CA and its assembly, although none of the currently identified compounds are used in the clinic for treatment of HIV infection. A specific interaction that is primarily present in pentameric interfaces in the HIV-1 capsid core was identified and is reported to be important for CA assembly. This is shown by multidisciplinary characterization of CA site-directed mutants using biochemical analysis of virus-like particle formation, transmission electron microscopy of *in vitro* assembly, crystallographic studies, and molecular dynamic simulations. The data are consistent with a model where a hydrogen bond between CA residues E28 and K30′ from neighboring N-terminal domains (CA_NTD_s) is important for CA pentamer interactions during core assembly. This pentamer-preferred interaction forms part of an N-terminal domain interface (NDI) pocket that is amenable to antiviral targeting.

## Abstract (precis)

Precise assembly and disassembly of the HIV-1 capsid core are key to the success of viral replication. The forces that govern capsid core formation and dissociation involve intricate interactions between pentamers and hexamers formed by HIV-1 CA. We identified one particular interaction between E28 of one CA and K30′ of the adjacent CA that appears more frequently in pentamers than in hexamers and that is important for capsid assembly. Targeting the corresponding site could lead to the development of antivirals which disrupt this interaction and affect capsid assembly.

## INTRODUCTION

**FIG 1:**
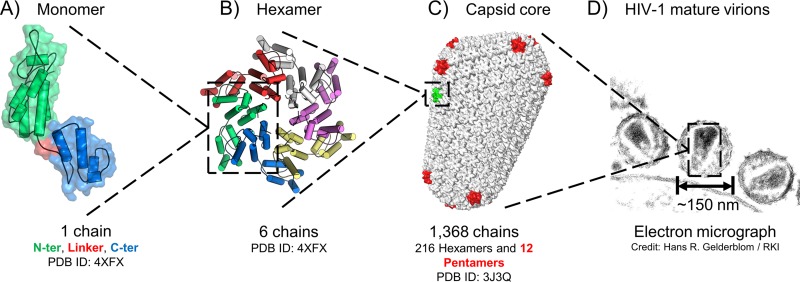
Structure and assembly of the HIV-1 capsid core. Capsid protein folds to form two domains connected by a flexible linker (A) and forms hexamers (B) and pentamers (red) in the mature core (C), which encloses the viral RNA and ultimately houses reverse transcription (D).

The HIV-1 capsid protein (CA) plays crucial roles in both early and late stages of the viral replication cycle (1). CA consists of 231 amino acids that fold into two distinct domains, the N-terminal domain (CA_NTD_) and the C-terminal domain (CA_CTD_), connected by a short linker. Multiple copies of CA monomers assemble around the viral genome to form the characteristic cone-shaped structure of the mature HIV-1 capsid core (2) (Fig. 1). The core is composed of ∼200 hexamers and precisely 12 pentamers. As with other icosahedral and fullerene structures, the 12 pentamers are required to form a closed core structure (3, 4).

During the early stages of HIV-1 replication, after viral and host cell membrane fusion, the capsid core is released into the cytoplasm, where it protects the viral RNA genome and protein components from degradation. Reverse transcription occurs in the core and is tightly coupled to the poorly understood process of capsid core disassembly, or uncoating (5–10). These events lead to the importation of the double-stranded viral DNA into the host cell nucleus, where it is integrated into the host DNA.

Several models of uncoating have been proposed (1), each supported by different experimental data. These models mainly differ in the timing of uncoating, which occurs immediately after entry (11, 12) or ∼30 mins postfusion (10) or during nuclear trafficking (13–16) or when the capsid core reaches the nuclear envelope (17). The uncoating process is highly complex; it affects reverse transcription (5, 13, 18–21) and nuclear import and integration of viral DNA (5, 16) and involves interactions between viral partners (matrix, reverse transcriptase, integrase, Vpr [11, 12]) and host partners (cyclophilin A [CypA]) (16), microtubules (22), dynein (23), kinesin (24), cleavage and polyadenylation specificity factor 6 (CPSF6) (25, 26), transportin 3 (TNPO3) (27), and nuclear pore proteins NUP358 and NUP153 (15), all regulated in a spatiotemporal fashion.

Uncoating not only occurs due to specific interactions with various partners but is also influenced by CA structure and flexibility and requires appropriate stability of the core itself (18). Intermolecular CA_NTD_/CA_NTD_ interactions are essential for the formation and stabilization of the hexameric (28) and pentameric (29) CA_NTD_ rings observed in assembled cores. CA_NTD_/CA_CTD_ interactions are not observed within each monomer but instead occur between neighboring monomers within a hexamer or pentamer (28, 29). As CA_NTD_/CA_NTD_ and CA_NTD_/CA_CTD_ interactions differ between neighboring monomers in the hexamer or pentamer (29–31), we will use the terms “hexameric interface” and “pentameric interface” to refer to them.

In contrast, CA_CTD_/CA_CTD_ interactions occur between monomers of neighboring hexamers (or pentamers). Mobility at these CA_CTD_/CA_CTD_ interfaces, which connect hundreds of hexamers and 12 pentamers, accommodates the curvature of the capsid core (28, 30). Additionally, nuclear magnetic resonance (NMR) and mutagenesis studies have described the linker connecting the CA_NTD_ and CA_CTD_ as highly flexible (32) and essential for proper assembly and stability of the core (33–35). Results of coarse-grained model simulations suggest that linker flexibility explains the polymorphism of CA assemblies (36) by enabling variable CA_NTD_/CA_CTD_ orientations.

**FIG 2:**
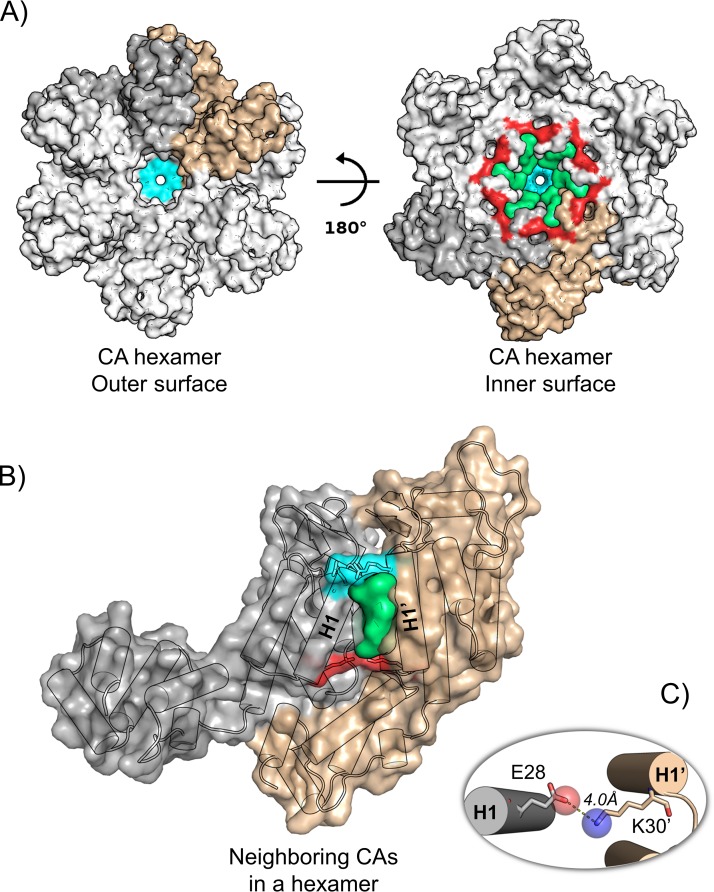
N-terminal domain interface (NDI) pocket. (A) The NDI pocket (in green) is located on the inner surface of the core, symmetrically encircling the 6-fold and 5-fold axes in hexamers and pentamers. (B) The pocket is formed at the interface between two neighboring CA_NTD_s. H1 and H1′ form the sides of the pocket, and it is capped at one end by two R18 sidechains (in cyan) and by an E28-K30′ interaction (in red) at the other end. (C) Close-up representation of the E28-K30′ interaction in this hexamer structure (PDB ID: 4XFX). The two sidechains are slightly too far apart to form a direct hydrogen bond.

Recent NMR studies (37) have shown that major variations in different *in vitro* CA assembly morphologies (tubes, sheets, and spheres) involve minor variations in the molecular structures of ordered segments, suggesting changes in the intermolecular CA_CTD_ dimerization interface and changes in the intramolecular helix-helix packing in the CA_NTD_. Additionally, the narrow end of the conical capsid core has been proposed to be a weak point for disassembly. Indeed, this area has a higher concentration of pentamers, which have been suggested to be less stable than hexamers due to the tighter positioning and greater electrostatic repulsion of the arginine 18 residues that form a tight ring at the center of hexamers and pentamers (29) (Fig. 2A). Mutagenesis studies have also highlighted the extreme genetic fragility of CA assembly, particularly that of the CA_NTD_ helices, which appear to be more sensitive than CA_CTD_ to mutations impacting the structure and stability of the CA hexamer assembly (19).

The multiple roles of CA in the virus replication cycle and its sensitivity to mutations have led to increases in efforts devoted to discovering antivirals targeting CA (38). This has led to the identification of several ligands (39), some of which impact virion maturation by inhibiting core assembly (CAP, benzodiazepines [BD], and benzimidazole [BM] compounds [40, 41]) whereas others impact the uncoating process (BI compounds [42]) and yet others affect both stages, as well as reverse transcription (PF74 [43, 44]). The effect of ligand binding on viral uncoating has been extensively studied, and it is not yet clear whether the effect is the result of an increase or decrease in the stability of CA assemblies. However, the binding of compounds to the capsid core may also prevent interactions with the host protein partners required for proper uncoating, as observed for BI-2 and PF74, whose shared binding sites overlap those of CPSF6 and NUP153 (45–47).

Identification of sensitive interactions at CA interfaces may aid the development of new antivirals targeting the stability of CA assemblies. In this study, we explored sites in capsid that differ between pentameric and hexameric environments to identify novel targets for the design of inhibitors that interfere with proper assembly of the HIV-1 core. The importance of a previously unreported interaction between neighboring CA monomers within pentamers is highlighted, and a novel site for design of new antivirals targeting core assembly was identified and characterized using biochemical analysis of virus-like particle (VLP) formation, transmission electron microscopy (TEM) of *in vitro* assembly, crystallographic studies, and molecular dynamic (MD) simulations.

## RESULTS

### Capsid interactions in hexamers versus pentamers.

Currently, two atomic models of the entire capsid core are available in the Protein Data Bank (PDB) archive (PDB identifiers [ID]: 3J3Q and 3J3Y) (31). Both were built using an integrative combination of computational techniques (molecular dynamics flexible fitting, molecular modeling, and all-atom molecular dynamics simulations) and experimental data from cryo-electron microscopy (cryo-EM), cryo-electron tomography (cryo-ET), X-ray crystallography, and NMR spectroscopy. Using these models, we analyzed the statistical prevalence of molecular interactions at the interface of neighboring CA molecules in pentameric or hexameric units (see Fig. S1 in the supplemental material). One interaction is significantly more frequent at the pentameric interface than at the hexameric interface: a hydrogen bond (H-bond) between the E28 of helix 1 (H1) in one CA_NTD_ and the K30′ in helix 1′ (H1′) of the neighboring CA_NTD_. This interaction is present in 86.7% of pentameric interfaces compared to 24.2% hexamer interfaces (Table 1). The distances between Cα atoms of E28 and K30′ in neighboring subunits in these structures are also smaller in pentamers than in hexamers. A similar trend is seen in 20 subnanometer cryo-ET structures from intact virions (30), showing closer proximity of E28 to K30′ Cα in pentamers than in hexamers (Table 1). This interaction is at the bottom of a pocket between CA residues from neighboring N-terminal domains (CA_NTD_s) (Fig. 2B and C). Here, we use the term “NDI pocket” to refer to this N-terminal domain interface pocket.

**TABLE 1:** H-bond and experimental Cα-Cα distances between E28 and K30′^*a*^

Method (PDB IDs)	No. of dimers	% dimers with E28∼K30′ H-bond	Cα-Cα distance (Å)
Modeling (3J3Q and 3J3Y)^*b*^			
Hexamer	2,412	24.20	14.45 (±2.02)
Pentamer	120	86.70	11.91 (±0.98)
			
Cryo-ET (5MCX → 5MCZ, 5MD0 → 5MD9, and 5MDA → 5MDG)^*c*^			
Hexamer	104		13.64 (±0.34)
Pentamer	5		10.05 (±0.04)

a H-bonds were identified using a distance threshold of 3.35 Å between E28 carboxyl oxygen and K30′ amine nitrogen.

b ΔCα-Cα (hexamer − pentamer) (Å), 2.54.

c ΔCα-Cα (hexamer − pentamer) (Å), 3.59.

10.1128/mBio.02858-18.1 FIG S1 Comparison of H-bond interaction frequencies at the CA monomer/monomer interfaces in the context of the hexamer or pentamer. (A) H-bond frequencies in hexamers (among 2,412 monomer/monomer interfaces) versus pentamers (among 112 interfaces). Interactions observed in at least 20% of dimers (within either hexamers or pentamers) are labeled. The E28∼K30′ interaction is underlined in red. (B) Subunits that form the H-bond interaction are shown in red for pentamers and green for hexamers. Download FIG S1, TIF file, 4.1 MB. Copyright © 2019 Craveur et al. 2019 Craveur et al. This content is distributed under the terms of the Creative Commons Attribution 4.0 International license.

Consistent with this observation, the interaction is not observed in the X-ray structure of the native wild-type (WT) CA in hexameric form (PDB ID: 4XFX) (48) or in any of the hexameric CA X-ray crystal structures available in the PDB, i.e., dehydrated, mutated, or bound to a ligand or peptide. An interaction between E28 and K30′ mediated by water molecules is observed in the X-ray crystal structure of the cross-linked hexamer (PDB ID: 3H47) (28) (see Table 5 in reference 48). One structural feature could explain the absence of this interaction in the X-ray crystal structures: the crystal lattice is flat and does not fully recapitulate the core’s characteristic curvature. This curvature is expected to impact the relative orientations of neighboring CA_NTD_s (30) within pentameric or hexameric assemblies (37) and to be more pronounced in the presence of pentamers (29, 31).

The only available X-ray crystal structure of a pentamer also does not show a hydrogen bond at this site. It is cross-linked through engineered cysteines at positions 21 and 22 in the H1 helix (PDB ID: 3P05) (29). The resulting disulfide bond is at the top of the NDI pocket, between position Cys21 of the H1 in one monomer and Cys22′ of the H1′ in the neighboring monomer. This engineered constraint brings E28 and K30′ closer at the end of the helices, requiring a reorientation of their side chains to avoid steric clashes.

### E28, K30, and R18 are highly conserved.

Residues E28, K30, and R18 are highly conserved, with 99.4%, 98.8%, and 99.4% sequence identity across the subtype reference sequences available in the Los Alamos National Laboratory HIV mutation browser (http://hivmut.org [49]). Only three isolates, *03GH173_06* (subtype *06_cpx*; GenBank accession number AB286851 [https://www.ncbi.nlm.nih.gov/nuccore/AB286851]), *nx2* (subtype *08_BC*; GenBank accession number HM067748 [https://www.ncbi.nlm.nih.gov/nuccore/HM067748]), and *A32989* (subtype *BF1*; GenBank accession number AF308491 [https://www.ncbi.nlm.nih.gov/nuccore/AF308491]) show substitutions at those positions with K30R, E28D/K30E, and R18K, respectively (see Table S1 in the supplemental material).

10.1128/mBio.02858-18.5 TABLE S1 Sequence alignment of CA isolates from different subtypes (positions 18 through 38) to the CA WT (*HXB2*). Download Table S1, TIF file, 0.7 MB. Copyright © 2019 Craveur et al. 2019 Craveur et al. This content is distributed under the terms of the Creative Commons Attribution 4.0 International license.

**FIG 3:**
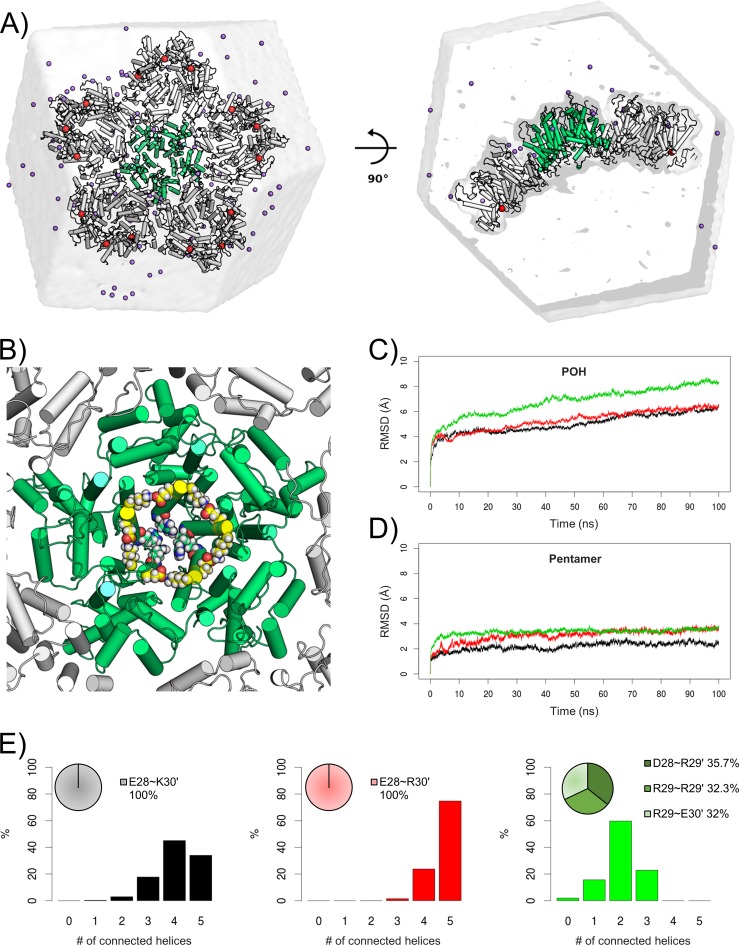
MD simulation for CA pentamers of hexamers (POH) from three isolates. (A) Dynamics of the pentamers (in green) of hexamers (in white) were simulated in a periodic dodecahedron box of water molecules. The charge of the system is neutralized with sodium counterions (purple spheres), and the curvature of the assembly is maintained with positional restraints on 15 Cα carbons (red spheres; see Materials and Methods). (B) In the starting structure of the WT (*HXB2*), a ring of H-bonds is formed by E28 and K30′, which connect five H1 helices (in yellow). (C and D) Backbone RMSD relative to starting structures for *HXB2* (black), *03GH173_06* (red), and *nx2* (green) for the entire POH (C) and the central pentamer (D). (E) Statistics on the number of hydrogen bonds formed between the ends of both helices H1 and H1′ (positions from 28 to 31) as monitored over the entire simulation. Pie charts identify the particular H-bonds being formed. Block-averaged statistics are shown in Fig. S3.

In order to determine whether similar interactions at the E28∼K30′ site are formed in other subtypes, 100-ns molecular dynamic (MD) simulations of pentamers of hexamers (POH; one pentamer surrounded by five hexamers) were performed for the WT (*HXB2*), *03GH173_06*, and *nx2* isolates. We extracted one POH from the WT core structure (PDB ID: 3J3Y) and used it as a template to model the POH of the isolates named above. The characteristic curvature was maintained by constraining the positions of three atoms in each hexamer (see Materials and Methods and Fig. 3A and B). The backbone root mean square deviations (RMSDs) computed over the trajectory for the central pentamer (Fig. 3D) and the surrounding hexamers show that stable conformations were reached quickly (after ∼10 ns) (Fig. S2). However, no equilibrium was reached for the overall POH complexes (Fig. 3C) even after 100 ns of simulation. These data indicate that the POH systems continue to evolve over the trajectories at the intersubunit interface.

10.1128/mBio.02858-18.2 FIG S2 Stability of the hexamers from the POH MD simulations. The evolution of the backbone RMSDs over time (indicated in nanoseconds [ns]) is shown for each capsid isolate compared to the starting conformation for each hexamer of the POH complex. Download FIG S2, TIF file, 4.3 MB. Copyright © 2019 Craveur et al. 2019 Craveur et al. This content is distributed under the terms of the Creative Commons Attribution 4.0 International license.

10.1128/mBio.02858-18.3 FIG S3 Block-averaged statistics on hydrogen bonds formed between helices H1 and H1′ for POH simulations from three isolates. Data represent statistics on the numbers of hydrogen bonds formed between helices H1 and H1′ (positions 28 to 31) as monitored in simulations of 25-ns increments from 1 to 100 ns (Fig. 3E shows statistics for the entire 100-ns simulation). Pie charts identify the residues forming H-bonds. Statistics for the *HXB2* isolate are shown in black, *03GH173_06* in red, and *nx2* in green. Download FIG S3, TIF file, 2.3 MB. Copyright © 2019 Craveur et al. 2019 Craveur et al. This content is distributed under the terms of the Creative Commons Attribution 4.0 International license.

Analysis of the H-bond interactions connecting the bottom of helices H1 and H1′ (from position 28 to position 31) reveals that for both the WT isolate (*HXB2*) and isolate *03GH173_06*, H-bonds between positions 28 and 30 (E28∼K30′ and E28∼R30′, respectively) participated in the formation of a closed ring of interactions in the pentamer (Fig. 3B and E; see also Fig. S3). For isolate *nx2*, a similar ring formation was never observed, but a larger set of H-bonds were found to be able to connect helices H1 and H1′. Interestingly, the latter isolate has 4 substitutions in positions 28 through 31 (Table S1), which add 5 additional charges and drastically decrease the inherent nonbonded potential energy of these residues compared to the results seen with the other isolates (−1,836.2 ± 26.2 kcal·mol^−1^ for the WT isolate, −2,611.0 ± 30.0 for isolate*03GH173_06*, and −3,219.2 ± 35.8 for isolate *nx2*).

### Effect of E28 and K30 on particle formation.

Previous mutagenesis and cell-based infectivity studies highlighted the importance of capsid residues E28 and K30 for virus replication. Rihn et al. showed that substitutions of K30 with shorter, oppositely charged (K30E) or uncharged (K30N) amino acids resulted in the production of noninfectious particles (19). However, no catastrophic structural changes are apparent in cryo-EM images of the WT and K30N viruses (personal communication). Similarly, the two E28A/E29A mutations resulted in the production of noninfectious virus. Studies of the E28A/E29A viral particle morphology performed using transmission electron microscopy (TEM) suggested normal levels of particle release. However, core formation was eliminated (20, 50), highlighting the finding that the reduction or loss of infectivity was directly linked to the lack of core assembly.

**FIG 4:**
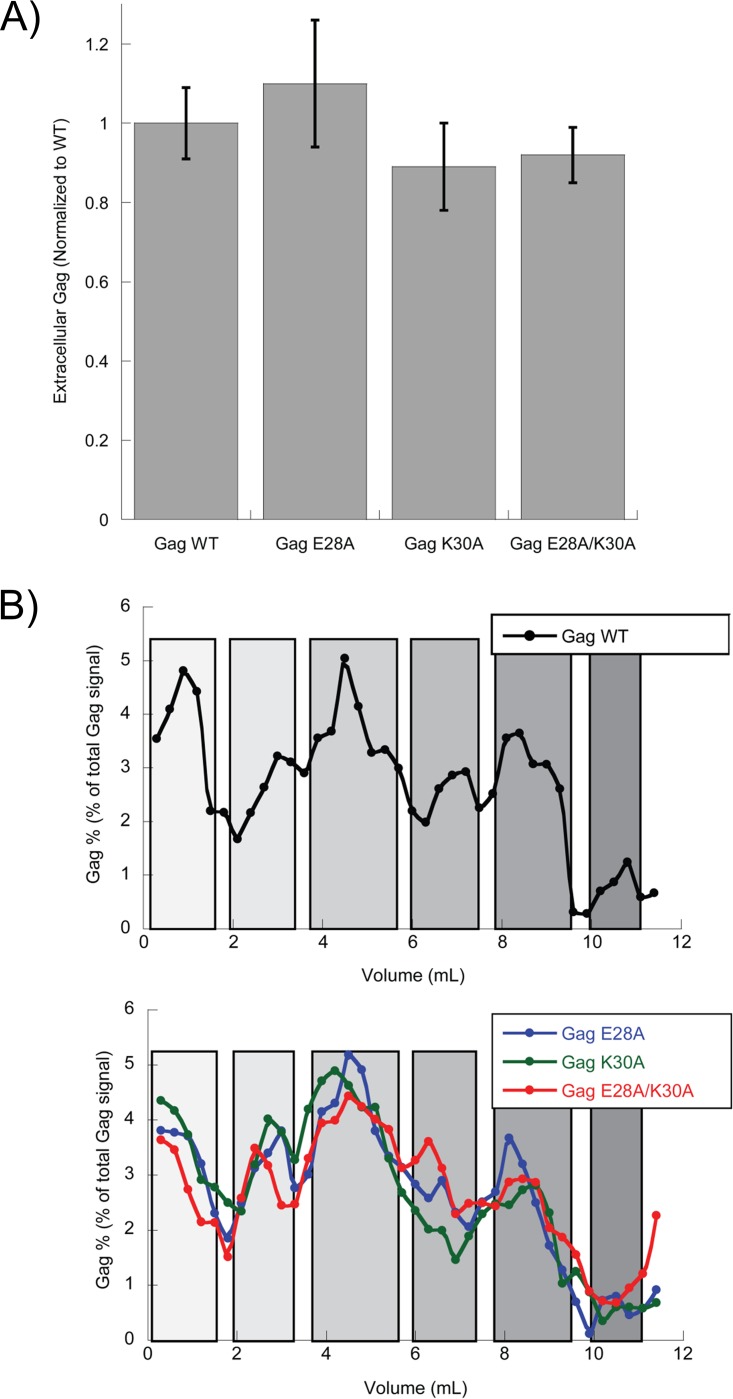
Gag mutations do not affect immature virus formation. (A) Graph of expression of extracellular Gag as a measure of VLP formation. All data have been normalized to Gag-WT. (B) WT and mutant Gag-containing complexes separated on a 10 to 60% sucrose gradient. Top of gradient is on left, and bottom on right. Gray boxes denote distinct intracellular Gag-containing populations described previously (49–51).

It is possible that E28 and K30 play a role in immature particle formation when CA is still part of the full-length Gag polyprotein. To test their effect on the formation of the HIV particle before maturation, E28A, K30A, and E28A/K30A mutations were introduced into a plasmid construct containing the HIV-1 Gag polyprotein (see Materials and Methods). Gag polyprotein alone can produce virus-like particles (VLPs) and produce intracellular Gag-containing complexes that sediment in a sucrose gradient identically to WT viral constructs (51–53). The three mutants displayed similar amounts of extracellular Gag 24 h posttransfection, indicating similar levels of VLP formation, and these mutations formed the intracellular Gag-containing complexes found in WT, indicating no defect in immature Gag particle formation or release (Fig. 4).

### Assembly of recombinant mutant CA proteins.

WT CA is known to assemble *in vitro* into long, hollow tubular structures. These tubes are composed of CA hexamers and are homogeneous in width with an external diameter of ∼55 nm but highly heterogeneous in length (4, 54, 55). It was previously shown that E28A/E29A reduces, but does not eliminate, CA assembly *in vitro* (50), producing cylinders similar to the WT CA at higher protein concentrations (∼15 mg/ml) but with severe attenuation of their production at lower protein concentrations (∼5 mg/ml).

Furthermore, R18A and R18A/N21A have been previously shown to impact the morphology of the *in vitro* CA assemblies by shifting the observed phenotypes to spheres, cones, spirals, and short capped cylinders (50). The R18A phenotype is thought to be the result of an increased frequency of pentameric interface formation in the assembling capsid lattice. The side chains of R18 create a strong positively charged pore in the middle of the CA_NTD_ rings that has been proposed to recruit nucleotides for importation into the core (56). The charges are brought closer together in the pentameric assembly than in the hexamer assembly, resulting in stronger electrostatic repulsion, which disfavors pentamers relative to hexamers (29). Further studies revealed that elimination of charge by replacement of R18 with a large hydrophobic residue (i.e., V, I, L, F) favored pentameric interface incorporation and induced assembly of spheres, presumably due to the stabilizing effect of hydrophobic contacts (29, 50, 57). With variation of the assembly conditions, those mutants also yielded cylinders, cones, and very large spheres (up to ∼2.5 µm in diameter), which collapsed and flattened (57) upon deposition on the EM grid. Most recently, the pocket composed of the six R18 residues from each CA monomer in a hexamer was shown to be the binding site for inositol hexaphosphate (IP6), which stabilizes hexamers and promotes DNA synthesis (58–61).

**FIG 5:**
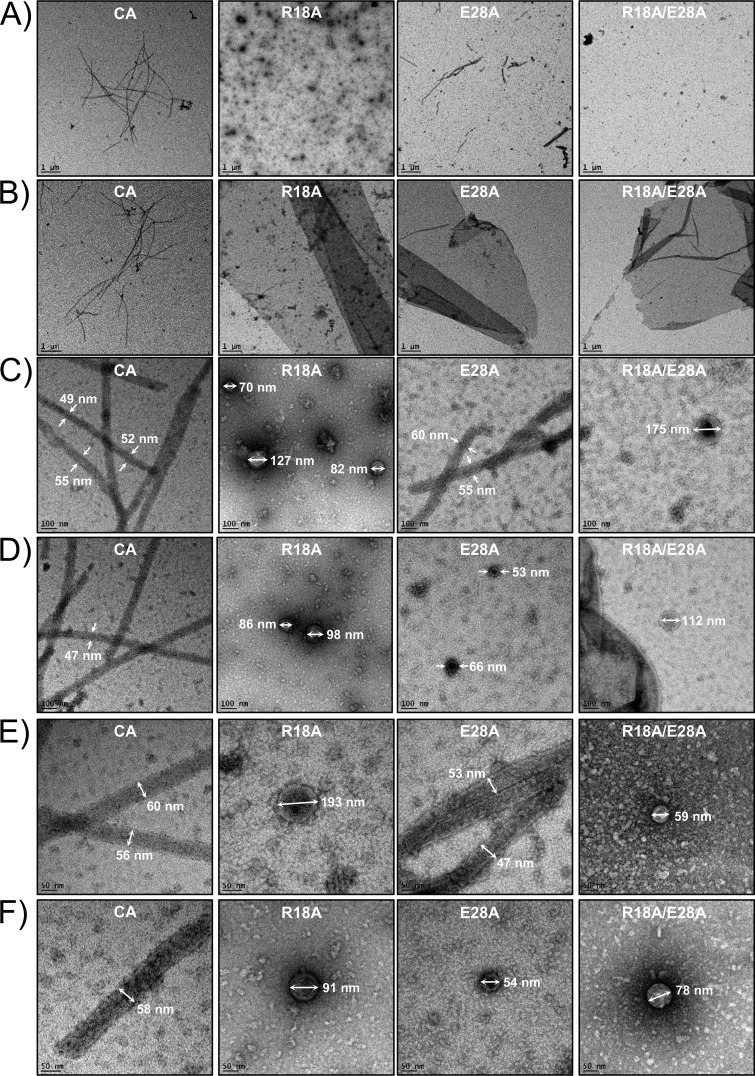
Effects of R18A, E28A, and R18A/E28A mutations on CA assembly. Images represent results of TEM analysis of CA mutant assemblies. Projection images were recorded at ×2,500 magnification (A to B), ×20,000 magnification (C and D), and ×40,000 magnification (E and F) from the corresponding samples as indicated. Scale bars are 1 µm in panels A and B, 100 nm in panels C and D, and 50 nm in panels E and F.

To assess the proposed role of the E28∼K30′ H-bond in CA assembly, we tested recombinant mutant CA proteins, harboring R18A, E28A, and R18A/E28A mutations, for cylinder formation *in vitro* (Table 2). WT and mutant CA proteins were assembled *in vitro* and analyzed by negative-stain TEM (Fig. 5). Consistent with previous reports, under experimental conditions WT CA formed hollow cylinders (or tubes) that were homogeneous in diameter (∼45 to 60 nm) but extremely heterogeneous in length. As expected, R18A mutant CA assembled efficiently, forming sheets and spheres that were highly variable in diameter (diameters ranged from 30 to 220 nm, occasionally achieving ∼400 nm), i.e., phenotypes that are consistent with the presence of pentameric assembly *in vitro* (Fig. 5). Notably, shortening the R18 side chain (R18G and R18A) should neither affect the overall structure of the CA protein nor impact its ability to form hexameric assemblies (56), since these are present in the observed morphologies.

**TABLE 2:** Self-assembly phenotypes of HIV-1 CA mutations *in vitro*

Protein	*In vitro* assembly	Phenotype	Interpretation^*a*^
CA	+ + +	Tubes	Hexamer >> pentamer
R18A	+ + +	Spheres, sheets	May stabilize pentamers
E28A	+ + (attenuation)	Tubes, spheres, sheets	Hexamer > pentamer
R18A/E28A	+ (attenuation)	Spheres, sheets	May stabilize pentamers

a +++, efficient *in vitro* assembly as compared to WT CA; ++, attenuated (3-6 fold decrease) *in vitro* assembly as compared to WT CA; +, severely attenuated (>6 fold decrease) *in vitro* assembly as compared to WT CA. Tubes and sheets are assumed to be formed by hexamers; spheres are assumed to also contain pentamers.

The E28A mutant formed tubes, although their assembly was attenuated in comparison to the WT CA; it also formed sheets and spheres (diameters ∼50 to 130 Å) (Fig. 5). The appearance of sheets and spheres may have been a consequence of enhancement of pentamer formation or of suppression of hexamer formation or of a combination of the two. The R18A/E28A double mutant also showed attenuated assembly of sheets and a small number of spheres (with diameters ranging between 30 and 210 nm). On the basis of these data, we suggest that the R18A mutation is primarily responsible for the phenotype and that E28A decreases the assembly efficiency. Residues R18 and E28 outlined the NDI pocket (Fig. 2B), but since they were separated by ∼14 Å, the observed differences in assembly were likely not related to the removal of a direct contact between R18 and E28.

### Crystal structures of CA mutants.

**FIG 6:**
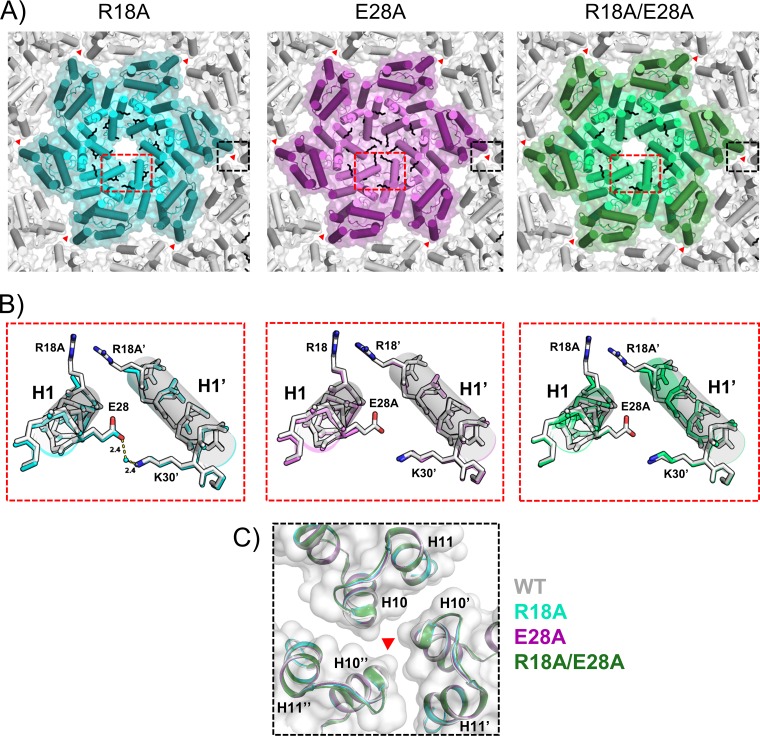
X-ray crystal structures of R18A, E28A, and R18A/E28A CA mutants compared to WT CA. (A) HIV-1 CA mutants R18A (CA_NTD_s in light cyan, CA_CTD_s in cyan), E28A (CA_NTD_s in light pink, CA_CTD_s in purple), and R18A/E28A (CA_NTD_s in light green, CA_CTD_s in green) form a hexamer that is very similar to WT CA (PDB ID: 4XFX). The side chains for the positions of interest (R18 or A18, E28 or A28, and K30′) are shown as black sticks. The 3-fold CA_CTD_ interfaces are indicated with red triangles. (B) Close-up views of red boxes in panel A. Two helices 1 (H1 and H1′), encompassing the NDI pocket, are very similar to the WT CA (shown in white). The side chains are shown for positions 18, 28, and 30′ only. For the R18A structure, a water molecule mediates an indirect H-bond interaction between E28 and K30′. (C) Close-up views of the 3-fold interfaces shown in panel A as black boxes. All CA mutant structures were aligned to the WT at the 3-fold axis (residue range, 195 through 221).

To address how the mutations mentioned above alter the structure of the CA protein, we crystallized and solved the crystal structures of R18A, E28A, or R18A/E28A capsid mutants. All structures were determined in the hexameric P6 space group with one molecule per asymmetric unit (see Table S2 and Materials and Methods). Overall, the mutant structures are very similar to the hexameric WT CA structure (RMSDs on backbone: 0.38 Å for R18A; 0.30 Å for E28A; 0.35 Å for R18A/E28A) (Fig. 6A). The side chains of all mutated residues were solvent exposed and not involved in significant intra- or intermolecular interactions in the CA crystal structures, thereby minimizing the chance of significant structural perturbations beyond the mutation site. This observation is further supported by buried surface area calculations: mutation of R18A in both single and double mutant structures resulted in an ∼10% decrease in the buried surface area at the intrahexamer interface (CA_NTD_/CA_NTD_ and CA_NTD_/CA_CTD_), while E28A has no significant effect. Interestingly, in addition to the localized changes and alteration of the intrahexamer interface, those mutations caused remote subtle side chain rearrangements that resulted in a 5% to 10% decrease of the buried surface area at the 2-fold interhexamer interface and a 30% to 60% decrease at the 3-fold interhexamer interface, as calculated using PISA software (62).

10.1128/mBio.02858-18.6 TABLE S2 Summary of X-ray data collection and refinement statistics. Download Table S2, TIF file, 1.1 MB. Copyright © 2019 Craveur et al. 2019 Craveur et al. This content is distributed under the terms of the Creative Commons Attribution 4.0 International license.

**FIG 7:**
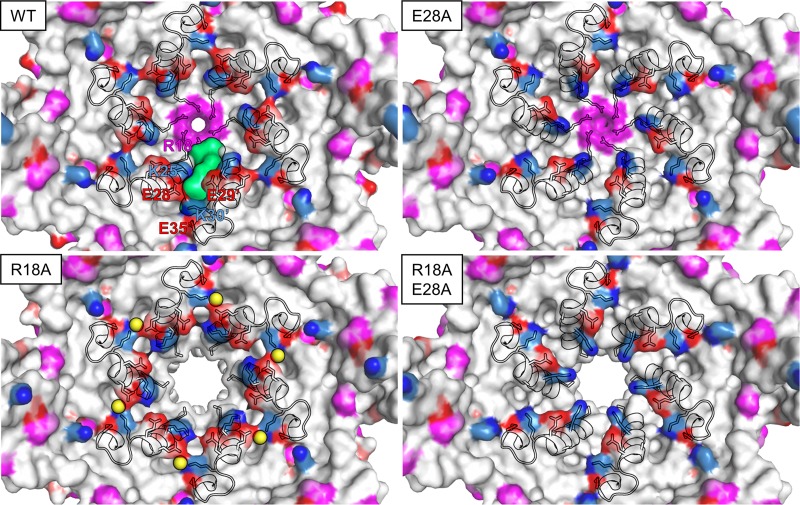
Charged amino acids at the NDI pocket in WT and mutant CA hexamers. Lysine is shown in blue, glutamate/aspartate in red, and arginine in purple (PDB ID: WT CA, 4XFX; structures reported here, 5W4O, 5W4P, and 5W4Q). One NDI pocket is highlighted in green (in the WT) with the surrounding charged amino acids labeled. H1 and the beginning of H2 are shown as cartoons. In R18A, the water molecules that bridge interaction between E28 and K30′ are shown as yellow spheres.

There was an indirect water-mediated H-bond interaction between E28 and K30′ in the R18A crystal structure (Fig. 6B); a similar interaction was observed in the crystal structure of the cross-linked hexamer (PDB ID: 3H47), while the E28A, R18A/E28A, and WT CA structures lacked this interaction. Furthermore, these mutations do not appear to have significantly affected the 3-fold interactions between hexamers (Fig. 6C). Overall, the R18A, E28A, and R18A/E28A X-ray crystal structures demonstrated that the mutations neither affected the folding of the CA protein nor dramatically altered the CA hexamer assembly. The structures also showed, as in previously determined structures of CA assemblies, that the E28∼K30′ interaction was part of a larger cluster of charged interactions at the site (Fig. 7). There appeared to be a facile reorganization of side chains to form salt bridges and alternate interaction in the hexamers, depending on the detailed configuration of the subunits. This is consistent with the alternative ring of interactions observed at this site in our MD simulations of the *nx2* isolate.

### MD Simulations of CA dimers.

**FIG 8:**
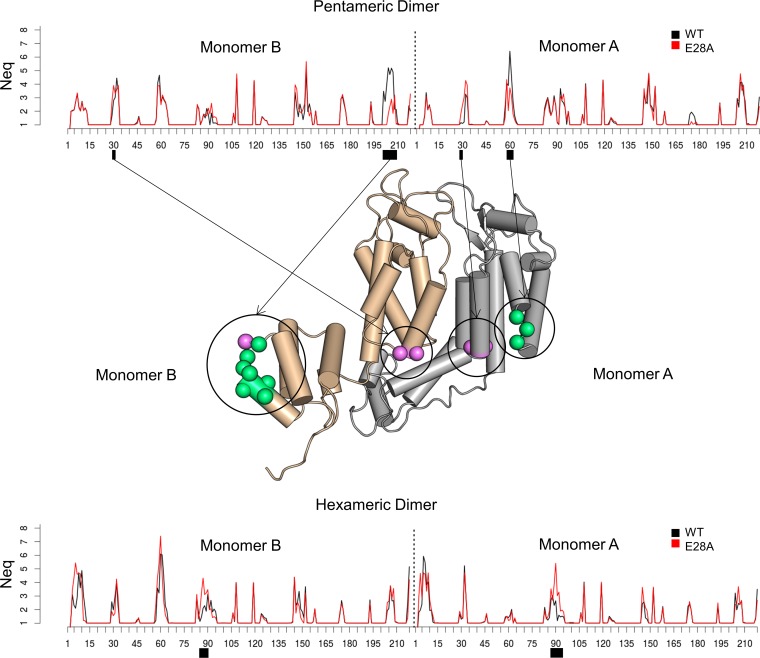
Backbone deformation of CA dimers comparing WT and E28A mutants in pentameric and hexameric conformations. The *N*_eq_ profiles of the two monomers of a pentameric dimer (top) and a hexameric dimer (bottom) are shown for the WT (in black) and the E28A mutant (in red). Values corresponding to the CA_CTD_ disordered region (positions 219 through 231) have been omitted for clarity. A cartoon representation of the pentameric dimer is shown in the center, and meaningful differences between both WT and mutant *N*_eq_ values are indicated with a purple sphere where the mutant has a higher value and with a green sphere where the WT has a higher value.

To observe whether the E28∼K30′ H-bond impacts the dynamics of the pentameric and hexameric interfaces, we performed two 100-ns MD simulations for the WT using two neighboring dimers extracted from the central pentamer of POH and a hexameric dimer extracted from the native CA X-ray hexamer crystal structure (PDB ID: 4XFX). Similar simulations were performed for the E28A mutant in both cases. By combining all the conformations extracted from the simulations at each frame (20,002 conformations for both WT and E28A), complete backbone deformation (*N*_eq_; see Materials and Methods) profiles were quantified for both the WT and mutant dimers (Fig. 8; see also Table S3).

10.1128/mBio.02858-18.7 TABLE S3 Atomistic MD simulations. Download Table S3, TIF file, 0.5 MB. Copyright © 2019 Craveur et al. 2019 Craveur et al. This content is distributed under the terms of the Creative Commons Attribution 4.0 International license.

Note that the dimers used for these computations did not correspond to the CA symmetric dimers (D_sym_) solved in solution NMR by Deshmukh et al. (PDB ID: 2M8L) (34). Here, the dimers were extracted from formed pentamers and hexamers. Consequently, monomers A and B of the dimer did not show the same flexibility as the positions involved at the interface, and the positions exposed to solvent were not the same for each monomer.

In pentameric dimers, positions surrounding the residues forming the H-bond (positions 30 and 31 from monomer A and positions 29 and 30 from monomer B) showed a slight increase of ∼1 *N*_eq_ unit in deformation in the mutant dimers. Also in the pentamers, the ends of helix H1 and helix H1′ (positions from 28 to 31) in the WT dimer were connected via the E28∼K30′ H-bond in 85.6% and 85.1% of the trajectories, respectively. In contrast, the ends of H1 and H1′ were almost never connected in the E28A mutant, with E28∼K30′ H-bonds formed in 0.1% and 0.4% of the trajectories and E29∼K30′ formed at only a few time steps. This suggests that the differences in *N*_eq_ results were related to enhanced deformation of the region from the absence of the E28∼K30′ H-bond.

Surprisingly, the major differences in deformation between WT and E28A occurred at distant regions of the sequences. In pentameric dimers, two regions, consisting of positions 59 to 61 of monomer A and positions 201 to 209 of monomer B, showed large decreases in deformation for the mutant. Residues 59 through 61 were positioned at the end of helix H3 close to the mutation site (∼5 Å), while positions 201 through 209 were positioned in a loop very distant from it (∼30 Å) and were largely accessible to solvent.

In hexameric dimers, more deformation was seen for the mutant in both monomers, around position 90, in the CypA loop at the top of CA_NTD_. Interestingly, the dynamics of this loop have already been described as determinant for binding of CypA (63), a host cell factor known to stabilize CA (64).

The intrinsic flexibility differences between the WT and the E28A variant suggest that destabilizing the E28-K30 interaction may affect the dynamics of the CA subunits in ways that may have implications for capsid assembly.

## DISCUSSION

CA residues R18, E28, and K30 are highly conserved among known HIV-1 isolates. They are at opposite ends of the N-terminal domain interface (or NDI) pocket formed by the ends of two symmetry-related helices (R18 and E28 in H1 and K30′ in H1′) from neighboring CAs within a pentameric or hexameric unit. R18 is at the “top” of the pocket, whereas E28 and K30′ are at the “floor” (Fig. 2). The NDI pockets seem to differ in intrapentamer versus intrahexamer interfaces.

In the available models of the HIV-1 cores, E28 and K30′ interact through an H-bond and form the floor of the NDI pocket in 87% of the intrapentamer and 24% of the intrahexamer interfaces. In contrast, interactions at the top of the pocket (R18) appear to be more important at intrahexameric interfaces, consistent with the loss of hexamer-containing tubes observed in our TEM studies with R18A. On the basis of the data, it is likely that both parts of the NDI pocket contribute to stabilization during assembly.

MD studies performed with WT and E28A CA dimers have identified a possible mechanism through which the E28∼K30′ interaction affects the global dynamics of CA during assembly. Loss of the E28∼K30′ interaction revealed changes in flexibility at residue positions proximal to E28 and K30′ (residues 59 to 61) and, interestingly, at a remote region (residues 201 to 209) as well (Fig. 8). The latter changes suggest that the E28A mutation at the interpentamer interface allosterically affects the interactions between the H1 and H1′ helices. Similar long-range interactions between remote CA regions have been previously described. Noviello et al. (65) reported that CA mutations H62A and H62F have no effects on the release of VLPs but result in aberrant core morphologies correlated to their infectivity defects. Remarkably, two compensatory mutations that improved infectivity were mapped to residue 208 (G208R and G208A). In the CA pentameric dimer, H62 is located behind the E28∼K30′ H-bond, and G208 is a part of the loop between helices H10 and H11. The authors proposed that H62 mutants alters the 3-fold CA_CTD_ interface and that mutation in residue 208 repositions helix H11 to accommodate the histidine 62 mutation.

Under physiological conditions, CA monomers interact via the 2-fold CA_CTD_/CA_CTD_ interface to form symmetric dimers (D_sym_) (66). Interactions between three D_sym_, around the 3-fold axis, form a trimer of dimers (TOD), which has been reported in coarse-grain and mathematical models as a building block of the CA hexameric lattice (36, 67). However, the recent coarse-grained simulation suggests that the addition of D_sym_ onto formed TODs represents the basic nucleation pathway rather than a simple aggregation of distinct TODs (35). As illustrated in Fig. 11 in reference 67 (or see Fig. S4A in the supplemental material), the pentamers can be viewed as an aberration in the TOD lattice, where two TODs overlap and share one D_sym_. In this context, pentamer formation into the CA lattice cannot result from the polymerization of distinct TODs but has to be the product of the addition of D_sym_ in the existing pentameric dimer (Fig. S4B and C).

10.1128/mBio.02858-18.4 FIG S4 Pentameric dimer and CA assembly. (A) In Fig. 11 in the article by M. Tsiang et al. (67), the pentamer (outlined by red pentagon) is presented as an aberration in the trimer of dimers (TOD) lattice. Two TODs share a single symmetric dimer (D_sym_) in the pentamer. (B and C) To incorporate a pentamer, a dimer with a pentameric interface (composed of monomers A and B in dark green) (B) has to interact with a D_sym_ (composed of monomers C and D in blue) (C). (D) We propose that positions 201 through 209 (shown as sticks) of monomer B (in red) confer specific flexibility that is required to incorporate the CA_CTD_ of monomer D of the D_sym_ and form the 3-fold interface. Download FIG S4, TIF file, 12.1 MB. Copyright © 2019 Craveur et al. 2019 Craveur et al. This content is distributed under the terms of the Creative Commons Attribution 4.0 International license.

Our results suggest that E28A affects neither the formation of the D_sym_ and the TOD nor their polymerization, as we observed tubes *in vitro* (Fig. 5). However, we showed that E28A affects the incorporation of pentamers. We thus propose that proper pentamer formation in the CA lattice requires specific flexibility of the H10/H11 loop at the free end of the CA_CTD_ (Fig. S4D). Moreover, we propose that the E28∼K30′ H-bond between the adjacent monomers in the pentameric dimer is the key to this flexibility.

Lemke et al. (41) previously identified the K30R substitution as a resistance mutation corresponding to the BM compounds, a family of CA assembly inhibitors that act primarily by blocking the assembly of mature conical capsids. The X-ray crystal structure of CA_NTD_ in complex with BM-4 (PDB ID: 4E92) showed binding in a hydrophobic pocket at the bottom of the CA_NTD_, just behind H1 and K30, that slightly reorganized the bottom of the helix bundle in the CA_NTD_. Interestingly, mutation of lysine to arginine, a similarly charged but longer amino acid, resulted in significant BM inhibitor resistance (4-fold for BM-2, 5-fold for BM-3) without reducing the binding of the compounds. The authors concluded that the “resistance conferred by these substitutions were not attributable to reduced inhibitor binding affinity, implying that they act via an indirect mechanism.” Regarding our study, it is tempting to speculate that the binding of BM compounds perturbs the formation of the E28∼K30′ H-bond and that the R30′ mutant positioning brings its charge closer to E28 in a range suitable for H-bond formation.

Finally, we propose the NDI pocket is an interesting target for the discovery and design of antiviral inhibitors. This pocket is (i) not subject to crucial binding site reorganization (it is located between helix bundles displaying low backbone deformations and mobility); (ii) located near the 6-fold axis channel, which could be interesting for designing compounds to disturb nucleotide recruitment through the pore; and (iii) capped at the top by two R18 residues and at the bottom by E28 and K30′, positions that have been identified previously and in the current study as important for core assembly. Moreover, the end of helix H3 (positions 58 and 59) forms the deepest part of the pocket and comprises the same loop as the neighboring H62, which is highly conserved, and its impact on capsid assembly has been well studied.

**FIG 9:**
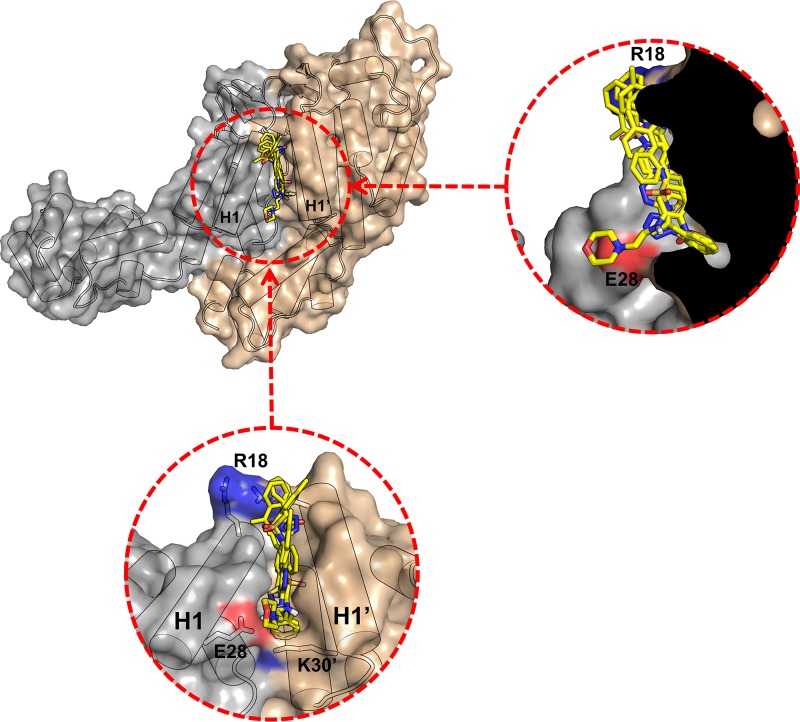
Top 5 compounds from a virtual screen of the NDI pocket. Docking results are shown as the superposition of five different molecules (yellow sticks) binding in the NDI pocket from the X-ray crystal structure of the native CA (PDB ID: 4XFX).

Given these observations, we performed a preliminary high-throughput virtual screen focusing on the NDI pocket using the FightAIDS@Home project (FA@H; http://fightaidsathome.scripps.edu/Capsid/index.html) in collaboration with IBM’s World Community Grid (https://www.worldcommunitygrid.org/). More than 1.6 million commercially available compounds have been used to target, *inter alia*, 20 conformations of the NDI pocket, selected from hexameric and pentameric interface assemblies. Preliminary results show that the NDI pocket is a plausible binding site for antiviral compounds (Fig. 9) from a molecular docking point of view. Evaluation and characterization of these compounds are the subjects of an ongoing independent study.

In conclusion, through biochemical, virological, TEM, crystallographic, and MD simulation analyses, we characterized interactions primarily present in pentameric interfaces in the HIV-1 capsid core and showed them to be important for assembly. Our data highlighted the importance of a novel N-terminal domain interface (NDI) pocket that is amenable to antiviral targeting.

## MATERIALS AND METHODS

### NDI pocket identification.

The NDI pocket was identified in the CA hexamer X-ray crystal structure (PDB ID: 4XFX [48]) using AutoSite (68). In this method, energetic grid points in space combined with a clustering algorithm are used to select high-affinity groups of points around the receptor. Clusters are then scored and ranked to provide a list of potential binding sites. Fifteen unique pockets were identified on the surface of the CA hexamer. The NDI pocket was scored in the top three pockets in terms of AutoSite score (AS score, 33.90; Nbr points, 186; RadGyr, 4.39; buriedness, 0.89).

### Modeling and MD simulations.

The CA pentamer and pentamers of hexamers (POH) models were constructed using MODELLER (69) as follows. The WT POH and CA pentamer coordinates were from PDB ID 3J3Y (pdb-bundle 21 chains c, d, e, f, and g). The WT POH was used as a template to model the POH of the *03GH173_06* and *nx2* isolates. Positions of specific atoms in the three POH systems were constrained to maintain the CA lattice curvature during the MD trajectory. The constrained atoms were the Cα of the proline 147 (P147) of monomers that are on the exterior ring of the POH and not in contact with the central pentamer (Fig. 3A). As P147 is a part of the linker and has no side chain, we assumed that the constraint does not drastically impact the intrinsic dynamics of either CA_NTD_ or CA_CTD_.

For the simulations of dimers, two pairs of monomers (chains c/d and chains f/g) were extracted from the pentamer and used as the templates to model two mutant E28A pentameric dimers by replacing the side chains of residues 28 in PyMOL (http://www.pymol.org/). Similarly, a pair of monomers was extracted from the X-ray crystal structure of the CA hexamer with PDB ID 4XFX. The missing parts of the structure (positions 6 to 10 and 222 to 231) were modeled using MODELLER (69). This hexameric dimer structure was used to model the mutant E28A hexameric dimer by replacing the side chains of residue 28 in PyMOL.

MD simulations were performed with GROMACS (70) using the AMBER99sb force field (71). Each structure was immersed in a periodic dodecahedral box using TIP3P water molecules (72) and neutralized with Na^+^ counter ions. The system was energetically minimized with a steepest-descent algorithm for 10,000 steps. The MD simulations were performed in an isotherm-isobar thermodynamics ensemble (NPT) with temperature fixed at 300 K and pressure at 1 bar. A run of 1 ns was performed to equilibrate the system using the Berendsen algorithm for temperature and pressure control (73). A production step of 100 ns or 200 ns was carried out using the Parrinello-Rahman algorithm (74) for temperature and pressure control, with coupling constants of T=0.1 ps and P=4 ps. All bond lengths were constrained with the LINCS algorithm (75), which allowed an integration step of 2 fs. The PME algorithm (76) was applied for long-range electrostatic interactions using a cutoff value of 1 nm for nonbonded interactions. Coordinates were saved every picosecond. Trajectory analyses were performed with GROMACS software and in-house R scripts.

Nonbonded interactions of the bottom of H1 pentamer helices were computed using the *g_energy* command from GROMACS software. The group composed of residues 28, 29, 30, and 31 in the five monomers was defined to compute their Lennard-Jones and Coulomb interactions with themselves and also with the rest of the proteins. The obtained values were summed for each frame and were averaged over the trajectory.

### CA backbone deformations and mobility analysis.

Backbone deformations were quantified using *N*_eq_ (77), a measure of structural variability. First, structural alphabet protein blocks (PBs), composed of 16 prototypes (78), were employed to classify local conformations. Each specific PB is characterized by the φ, ψ dihedral angles of five consecutive residues. The 16 PBs give a reasonable approximation of all local protein 3D structures (79). PB assignment was carried out using PBxplore (80) for each CA monomer from both capsid core models. From this description, we computed the *N*_eq_ (77), a statistical measurement similar to entropy that represents the average number of PBs that a residue may adopt at a given position. It is calculated as follows:

$$N_{\text{eq}}=\text{exp}\left[-\overset{16}{\underset{\text{PB}=1}{\sum }}f_{\text{PB}}ln\left(f_{\text{PB}}\right)\right]$$

where *f*_PB_ is the frequency of a particular PB at the position of interest and the sum is performed over all 16 PBs. An *N*_eq_ value of 1 indicates a rigid region where only one type of PB is observed, while a value of 16 indicates a totally flexible region with equal frequencies for all 16 states. Values for disordered parts of proteins are typically close to or higher than 8.

### pGagP6-CTE.

pGagP6-constitutive export element (pGagP6-CTE) (described previously [81]) was produced by replacing the BH10 HIV sequence with the NL4-3 lb/in^2^ signal and Gag protein through the P6 domain (deleting the frame-shifted protease containing extended polyprotein) using Gibson cloning. The final plasmid contained the viral Gag sequence under the control of a cytomegalovirus (CMV) promoter, and nuclear export was achieved via a CTE in the 3′ untranslated region (3′-UTR) of the resulting RNA. Mutations were introduced into pGagP6-CTE using an NEB Q5 site-directed mutagenesis kit (E0554S), and all sequences were verified by analysis of capillary sequencing reactions.

### Transfection and VLP formation.

pGag-CTE and mutations were transfected into HEK293T cells 24 h postseeding using polyethylenimine (PEI) as described previously (82). The supernatant (10 ml) was collected for each sample 24 h posttransfection, and cells were harvested via trypsin digestion.

### Sucrose gradient centrifugation and Gag detection.

Expression of Gag-containing complexes was assayed as previously described (51, 53) with a few modifications. Gag-expressing cells were lysed in lysis buffer A (10 mM HEPES [pH 7.5], 10 mM NaCl, 1 mM magnesium acetate, 0.85% octyl-β-d-glucopyranoside) for 10 min on ice, and the lysates were spun at 500 × g for 5 min to clear insoluble nuclei. The lysates were then loaded onto the top of an 11-ml 10% to 60% sucrose gradient and spun in the ultracentrifuge using a Beckman Coulter SW41Ti rotor at 35,000 rpm for 3 h. Centrifuged samples were separated into 300-µl aliquots. A 100-µl volume of each fraction was added to 200 µl distilled water (dH_2_O), and all fractions were subsequently blotted onto low-fluorescence polyvinylidene difluoride (PVDF) membranes (Bio-Rad) using a microsample filtration manifold (Schleider & Schuell). On a separate membrane, serial dilutions of the VLP-containing supernatant collected as described above were also loaded onto PVDF membranes. All membranes were then subjected to standard Western blotting using a fluorescently labeled α-P24 antibody (Millipore MAB880-AF). Gag signal was detected using a Bio-Rad Versadoc 4000MP system and quantified using Image Studio 5.2.

### Design, expression, and purification of CA mutant proteins.

R18A, E28A, and R18A/E28A CA mutants were based on a pET11a construct (48). Mutations were introduced using overlap extension PCR cloning and verified by DNA sequencing. Mutant CA proteins were expressed and purified as previously described (48, 83).

### TEM assembly of CA mutants.

WT CA and CA mutants (R18A, E28A, and R18A/E28A) were assembled at 150 µM in buffer containing 1 M NaCl and 50 mM Tris-HCl (pH 8.1) at 37°C for 1 h. A 5-µl volume of each sample was adsorbed during 5 min on grids coated with colloidal carbon made hydrophilic by glow discharge for 45 s. After that step, excess fluid was removed, and grids were washed with water, fixed in a drop of 2% uranyl acetate, and dried before visualization using a JEOL JEM 1400 transmission electron microscope at magnifications of ×2,500, ×20,000, and ×40,000. Each reaction was repeated at least three times.

### Crystallographic structures of CA mutants.

Crystals of the CA mutant proteins grew at 18°C in hanging drops, containing 2 to 5 mg/ml of protein, 6% to 9% polyethylene glycol (PEG) 3350, 2% to 6% glycerol, sodium iodide, and sodium cacodylate. Hexagonal plate-like crystals appeared after 5 days, and crystal growth was completed over 2 weeks. Crystals were briefly dipped in paraffin oil before cryo-cooling in liquid nitrogen was performed.

Data were collected on a Dectris Eiger-16m detector at Advanced Photon Source (APS) beamline 23-ID-B, Argonne National Laboratory. Datasets were collected and processed using XDS (84). The data were examined for the presence of systematic absences. However, no characteristic patterns were observed. Thus, the crystals were indexed in hexagonal space group P6 with one CA molecule in the asymmetric unit. No twinning was present, as determined by the use of either POINTLESS (85) or XTRIAGE (86). Space group and twinning were also verified in ZANUDA (62). Initial phases were obtained by molecular replacement via PHASER (62) using WT CA (PDB ID: 4XFX) as the starting model. Several rounds of iterative model building and refinement were carried out using Coot (87) and PHENIX (86), REFMAC (62, 88), or PDBREDO (https://pdb-redo.eu/). Structure validation of final models was performed with MOLPROBITY (http://molprobity.biochem.duke.edu/). Accessible and buried surface areas were calculated using PISA (62). The figures showing structural information were generated in PyMOL (http://www.pymol.org/) and CCP4MG (62).

### Data availability.

Structure factors and coordinates have been submitted to the RCSB Protein Data Bank (PDB) for the R18A, E28A, and R18A/E28A CA X-ray crystal structures (PDB IDs: 5W4O, 5W4P, and 5W4Q, respectively).

## References

[1] Campbell EM, Hope TJ 2015 HIV-1 capsid: the multifaceted key player in HIV-1 infection. Nat Rev Microbiol 13:471–483. doi:10.1038/nrmicro3503. 26179359 PMC4876022

[2] Briggs JA, Wilk T, Welker R, Krausslich HG, Fuller SD 2003 Structural organization of authentic, mature HIV-1 virions and cores. EMBO J 22:1707–1715. doi:10.1093/emboj/cdg143. 12660176 PMC152888

[3] Ganser BK, Li S, Klishko VY, Finch JT, Sundquist WI 1999 Assembly and analysis of conical models for the HIV-1 core. Science 283:80–83. doi:10.1126/science.283.5398.80. 9872746

[4] Li S, Hill CP, Sundquist WI, Finch JT 2000 Image reconstructions of helical assemblies of the HIV-1 CA protein. Nature 407:409–413. doi:10.1038/35030177. 11014200

[5] Ambrose Z, Aiken C 2014 HIV-1 uncoating: connection to nuclear entry and regulation by host proteins. Virology 454-455:371–379. doi:10.1016/j.virol.2014.02.004. 24559861 PMC3988234

[6] Aiken C 2006 Viral and cellular factors that regulate HIV-1 uncoating. Curr Opin HIV AIDS 1:194–199. doi:10.1097/01.COH.0000221591.11294.c1. 19372808

[7] Matreyek KA, Engelman A 2013 Viral and cellular requirements for the nuclear entry of retroviral preintegration nucleoprotein complexes. Viruses 5:2483–2511. doi:10.3390/v5102483. 24103892 PMC3814599

[8] Francis AC, Marin M, Shi J, Aiken C, Melikyan GB 2016 Time-resolved imaging of single HIV-1 uncoating in vitro and in living cells. PLoS Pathog 12:e1005709. doi:10.1371/journal.ppat.1005709. 27322072 PMC4913920

[9] Cosnefroy O, Murray PJ, Bishop KN 2016 HIV-1 capsid uncoating initiates after the first strand transfer of reverse transcription. Retrovirology 13:58. doi:10.1186/s12977-016-0292-7. 27549239 PMC4994286

[10] Mamede JI, Cianci GC, Anderson MR, Hope TJ 2017 Early cytoplasmic uncoating is associated with infectivity of HIV-1. Proc Natl Acad Sci U S A 114:E7169–E7178. doi:10.1073/pnas.1706245114. 28784755 PMC5576815

[11] Miller MD, Farnet CM, Bushman FD 1997 Human immunodeficiency virus type 1 preintegration complexes: studies of organization and composition. J Virol 71:5382–5390. 9188609 10.1128/jvi.71.7.5382-5390.1997 PMC191777

[12] Fassati A, Goff SP 2001 Characterization of intracellular reverse transcription complexes of human immunodeficiency virus type 1. J Virol 75:3626–3635. doi:10.1128/JVI.75.8.3626-3635.2001. 11264352 PMC114854

[13] Hulme AE, Perez O, Hope TJ 2011 Complementary assays reveal a relationship between HIV-1 uncoating and reverse transcription. Proc Natl Acad Sci U S A 108:9975–9980. doi:10.1073/pnas.1014522108. 21628558 PMC3116424

[14] Hulme AE, Kelley Z, Foley D, Hope TJ 2015 Complementary assays reveal a low level of CA associated with nuclear HIV-1 viral complexes. J Virol 89:5350–5361. doi:10.1128/JVI.00476-15. 25741002 PMC4442523

[15] Di Nunzio F, Danckaert A, Fricke T, Perez P, Fernandez J, Perret E, Roux P, Shorte S, Charneau P, Diaz-Griffero F, Arhel NJ 2012 Human nucleoporins promote HIV-1 docking at the nuclear pore, nuclear import and integration. PLoS One 7:e46037. doi:10.1371/journal.pone.0046037. 23049930 PMC3457934

[16] Schaller T, Ocwieja KE, Rasaiyaah J, Price AJ, Brady TL, Roth SL, Hue S, Fletcher AJ, Lee K, KewalRamani VN, Noursadeghi M, Jenner RG, James LC, Bushman FD, Towers GJ 2011 HIV-1 capsid-cyclophilin interactions determine nuclear import pathway, integration targeting and replication efficiency. PLoS Pathog 7:e1002439. doi:10.1371/journal.ppat.1002439. 22174692 PMC3234246

[17] Arhel NJ, Souquere-Besse S, Munier S, Souque P, Guadagnini S, Rutherford S, Prevost MC, Allen TD, Charneau P 2007 HIV-1 DNA Flap formation promotes uncoating of the pre-integration complex at the nuclear pore. EMBO J 26:3025–3037. doi:10.1038/sj.emboj.7601740. 17557080 PMC1894778

[18] Forshey BM, von Schwedler U, Sundquist WI, Aiken C 2002 Formation of a human immunodeficiency virus type 1 core of optimal stability is crucial for viral replication. J Virol 76:5667–5677. doi:10.1128/JVI.76.11.5667-5677.2002. 11991995 PMC137032

[19] Rihn SJ, Wilson SJ, Loman NJ, Alim M, Bakker SE, Bhella D, Gifford RJ, Rixon FJ, Bieniasz PD 2013 Extreme genetic fragility of the HIV-1 capsid. PLoS Pathog 9:e1003461. doi:10.1371/journal.ppat.1003461. 23818857 PMC3688543

[20] von Schwedler UK, Stray KM, Garrus JE, Sundquist WI 2003 Functional surfaces of the human immunodeficiency virus type 1 capsid protein. J Virol 77:5439–5450. doi:10.1128/JVI.77.9.5439-5450.2003. 12692245 PMC153941

[21] Xu H, Franks T, Gibson G, Huber K, Rahm N, De Castillia C, Luban J, Aiken C, Watkins S, Sluis-Cremer N, Ambrose Z 2013 Evidence for biphasic uncoating during HIV-1 infection from a novel imaging assay. Retrovirology 10:70. doi:10.1186/1742-4690-10-70. 23835323 PMC3716918

[22] Sabo Y, Walsh D, Barry DS, Tinaztepe S, de Los Santos K, Goff SP, Gundersen GG, Naghavi MH 2013 HIV-1 induces the formation of stable microtubules to enhance early infection. Cell Host Microbe 14:535–546. doi:10.1016/j.chom.2013.10.012. 24237699 PMC3855456

[23] Pawlica P, Berthoux L 2014 Cytoplasmic dynein promotes HIV-1 uncoating. Viruses 6:4195–4211. doi:10.3390/v6114195. 25375884 PMC4246216

[24] Lukic Z, Dharan A, Fricke T, Diaz-Griffero F, Campbell EM 2014 HIV-1 uncoating is facilitated by dynein and kinesin 1. J Virol 88:13613–13625. doi:10.1128/JVI.02219-14. 25231297 PMC4248982

[25] Lee K, Ambrose Z, Martin TD, Oztop I, Mulky A, Julias JG, Vandegraaff N, Baumann JG, Wang R, Yuen W, Takemura T, Shelton K, Taniuchi I, Li Y, Sodroski J, Littman DR, Coffin JM, Hughes SH, Unutmaz D, Engelman A, KewalRamani VN 2010 Flexible use of nuclear import pathways by HIV-1. Cell Host Microbe 7:221–233. doi:10.1016/j.chom.2010.02.007. 20227665 PMC2841689

[26] Rasaiyaah J, Tan CP, Fletcher AJ, Price AJ, Blondeau C, Hilditch L, Jacques DA, Selwood DL, James LC, Noursadeghi M, Towers GJ 2013 HIV-1 evades innate immune recognition through specific cofactor recruitment. Nature 503:402–405. doi:10.1038/nature12769. 24196705 PMC3928559

[27] Valle-Casuso JC, Di Nunzio F, Yang Y, Reszka N, Lienlaf M, Arhel N, Perez P, Brass AL, Diaz-Griffero F 2012 TNPO3 is required for HIV-1 replication after nuclear import but prior to integration and binds the HIV-1 core. J Virol 86:5931–5936. doi:10.1128/JVI.00451-12. 22398280 PMC3347269

[28] Pornillos O, Ganser-Pornillos BK, Kelly BN, Hua Y, Whitby FG, Stout CD, Sundquist WI, Hill CP, Yeager M 2009 X-ray structures of the hexameric building block of the HIV capsid. Cell 137:1282–1292. doi:10.1016/j.cell.2009.04.063. 19523676 PMC2840706

[29] Pornillos O, Ganser-Pornillos BK, Yeager M 2011 Atomic-level modelling of the HIV capsid. Nature 469:424–427. doi:10.1038/nature09640. 21248851 PMC3075868

[30] Mattei S, Glass B, Hagen WJ, Krausslich HG, Briggs JA 2016 The structure and flexibility of conical HIV-1 capsids determined within intact virions. Science 354:1434–1437. doi:10.1126/science.aah4972. 27980210

[31] Zhao G, Perilla JR, Yufenyuy EL, Meng X, Chen B, Ning J, Ahn J, Gronenborn AM, Schulten K, Aiken C, Zhang P 2013 Mature HIV-1 capsid structure by cryo-electron microscopy and all-atom molecular dynamics. Nature 497:643–646. doi:10.1038/nature12162. 23719463 PMC3729984

[32] Shin R, Tzou YM, Krishna NR 2011 Structure of a monomeric mutant of the HIV-1 capsid protein. Biochemistry 50:9457–9467. doi:10.1021/bi2011493. 21995733 PMC3210698

[33] Jiang J, Ablan SD, Derebail S, Hercik K, Soheilian F, Thomas JA, Tang S, Hewlett I, Nagashima K, Gorelick RJ, Freed EO, Levin JG 2011 The interdomain linker region of HIV-1 capsid protein is a critical determinant of proper core assembly and stability. Virology 421:253–265. doi:10.1016/j.virol.2011.09.012. 22036671 PMC3573886

[34] Deshmukh L, Schwieters CD, Grishaev A, Ghirlando R, Baber JL, Clore GM 2013 Structure and dynamics of full-length HIV-1 capsid protein in solution. J Am Chem Soc 135:16133–16147. doi:10.1021/ja406246z. 24066695 PMC3946434

[35] Grime JM, Dama JF, Ganser-Pornillos BK, Woodward CL, Jensen GJ, Yeager M, Voth GA 2016 Coarse-grained simulation reveals key features of HIV-1 capsid self-assembly. Nat Commun 7:11568. doi:10.1038/ncomms11568. 27174390 PMC4869257

[36] Qiao X, Jeon J, Weber J, Zhu F, Chen B 2015 Mechanism of polymorphism and curvature of HIV capsid assemblies probed by 3D simulations with a novel coarse grain model. Biochim Biophys Acta 1850:2353–2367. doi:10.1016/j.bbagen.2015.08.017. 26318016

[37] Lu JX, Bayro MJ, Tycko R 2016 Major variations in HIV-1 capsid assembly morphologies involve minor variations in molecular structures of structurally ordered protein segments. J Biol Chem 291:13098–13112. doi:10.1074/jbc.M116.720557. 27129282 PMC4933226

[38] Tedbury PR, Freed EO 2015 HIV-1 gag: an emerging target for antiretroviral therapy. Curr Top Microbiol Immunol 389:171–201. doi:10.1007/82_2015_436. 25731773 PMC6941199

[39] Thenin-Houssier S, Valente ST 2016 HIV-1 capsid inhibitors as antiretroviral agents. Curr HIV Res 14:270–282. doi:10.2174/1570162X14999160224103555. 26957201 PMC4785820

[40] Tang C, Loeliger E, Kinde I, Kyere S, Mayo K, Barklis E, Sun Y, Huang M, Summers MF 2003 Antiviral inhibition of the HIV-1 capsid protein. J Mol Biol 327:1013–1020. doi:10.1016/S0022-2836(03)00289-4. 12662926

[41] Lemke CT, Titolo S, von Schwedler U, Goudreau N, Mercier JF, Wardrop E, Faucher AM, Coulombe R, Banik SS, Fader L, Gagnon A, Kawai SH, Rancourt J, Tremblay M, Yoakim C, Simoneau B, Archambault J, Sundquist WI, Mason SW 2012 Distinct effects of two HIV-1 capsid assembly inhibitor families that bind the same site within the N-terminal domain of the viral CA protein. J Virol 86:6643–6655. doi:10.1128/JVI.00493-12. 22496222 PMC3393593

[42] Lamorte L, Titolo S, Lemke CT, Goudreau N, Mercier JF, Wardrop E, Shah VB, von Schwedler UK, Langelier C, Banik SS, Aiken C, Sundquist WI, Mason SW 2013 Discovery of novel small-molecule HIV-1 replication inhibitors that stabilize capsid complexes. Antimicrob Agents Chemother 57:4622–4631. doi:10.1128/AAC.00985-13. 23817385 PMC3811413

[43] Blair WS, Pickford C, Irving SL, Brown DG, Anderson M, Bazin R, Cao J, Ciaramella G, Isaacson J, Jackson L, Hunt R, Kjerrstrom A, Nieman JA, Patick AK, Perros M, Scott AD, Whitby K, Wu H, Butler SL 2010 HIV capsid is a tractable target for small molecule therapeutic intervention. PLoS Pathog 6:e1001220. doi:10.1371/journal.ppat.1001220. 21170360 PMC3000358

[44] Shi J, Zhou J, Shah VB, Aiken C, Whitby K 2011 Small-molecule inhibition of human immunodeficiency virus type 1 infection by virus capsid destabilization. J Virol 85:542–549. doi:10.1128/JVI.01406-10. 20962083 PMC3014163

[45] Matreyek KA, Yucel SS, Li X, Engelman A 2013 Nucleoporin NUP153 phenylalanine-glycine motifs engage a common binding pocket within the HIV-1 capsid protein to mediate lentiviral infectivity. PLoS Pathog 9:e1003693. doi:10.1371/journal.ppat.1003693. 24130490 PMC3795039

[46] Fricke T, Buffone C, Opp S, Valle-Casuso J, Diaz-Griffero F 11 12 2014 BI-2 destabilizes HIV-1 cores during infection and prevents binding of CPSF6 to the HIV-1 capsid. Retrovirology doi:10.1186/s12977-014-0120-x. PMC4271331 25496772

[47] Price AJ, Jacques DA, McEwan WA, Fletcher AJ, Essig S, Chin JW, Halambage UD, Aiken C, James LC 2014 Host cofactors and pharmacologic ligands share an essential interface in HIV-1 capsid that is lost upon disassembly. PLoS Pathog 10:e1004459. doi:10.1371/journal.ppat.1004459. 25356722 PMC4214760

[48] Gres AT, Kirby KA, KewalRamani VN, Tanner JJ, Pornillos O, Sarafianos SG 2015 STRUCTURAL VIROLOGY. X-ray crystal structures of native HIV-1 capsid protein reveal conformational variability. Science 349:99–103. doi:10.1126/science.aaa5936. 26044298 PMC4584149

[49] Davey NE, Satagopam VP, Santiago-Mozos S, Villacorta-Martin C, Bharat TA, Schneider R, Briggs JA 2014 The HIV mutation browser: a resource for human immunodeficiency virus mutagenesis and polymorphism data. PLoS Comput Biol 10:e1003951. doi:10.1371/journal.pcbi.1003951. 25474213 PMC4256008

[50] Ganser-Pornillos BK, von Schwedler UK, Stray KM, Aiken C, Sundquist WI 2004 Assembly properties of the human immunodeficiency virus type 1 CA protein. J Virol 78:2545–2552. doi:10.1128/JVI.78.5.2545-2552.2004. 14963157 PMC369201

[51] Robinson BA, Reed JC, Geary CD, Swain JV, Lingappa JR 2014 A temporospatial map that defines specific steps at which critical surfaces in the Gag MA and CA domains act during immature HIV-1 capsid assembly in cells. J Virol 88:5718–5741. doi:10.1128/JVI.03609-13. 24623418 PMC4019110

[52] Dooher JE, Schneider BL, Reed JC, Lingappa JR 2007 Host ABCE1 is at plasma membrane HIV assembly sites and its dissociation from Gag is linked to subsequent events of virus production. Traffic 8:195–211. doi:10.1111/j.1600-0854.2006.00524.x. 17233757 PMC1865004

[53] Lingappa JR, Hill RL, Wong ML, Hegde RS 1997 A multistep, ATP-dependent pathway for assembly of human immunodeficiency virus capsids in a cell-free system. J Cell Biol 136:567–581. doi:10.1083/jcb.136.3.567. 9024688 PMC2134302

[54] Ehrlich LS, Agresta BE, Carter CA 1992 Assembly of recombinant human immunodeficiency virus type 1 capsid protein in vitro. J Virol 66:4874–4883. 1629958 10.1128/jvi.66.8.4874-4883.1992 PMC241323

[55] Gross I, Hohenberg H, Krausslich HG 1997 In vitro assembly properties of purified bacterially expressed capsid proteins of human immunodeficiency virus. Eur J Biochem 249:592–600. doi:10.1111/j.1432-1033.1997.t01-1-00592.x. 9370371

[56] Jacques DA, McEwan WA, Hilditch L, Price AJ, Towers GJ, James LC 10 8 2016 HIV-1 uses dynamic capsid pores to import nucleotides and fuel encapsidated DNA synthesis. Nature 536:349–353. doi:10.1038/nature19098. 27509857 PMC4998957

[57] Ganser-Pornillos BK, Cheng A, Yeager M 2007 Structure of full-length HIV-1 CA: a model for the mature capsid lattice. Cell 131:70–79. doi:10.1016/j.cell.2007.08.018. 17923088

[58] Mallery DL, Marquez CL, McEwan WA, Dickson CF, Jacques DA, Anandapadamanaban M, Bichel K, Towers GJ, Saiardi A, Bocking T, James LC 2018 IP6 is an HIV pocket factor that prevents capsid collapse and promotes DNA synthesis. Elife 7:e35335. doi:10.7554/eLife.35335. 29848441 PMC6039178

[59] Obr M, Krausslich HG 2018 The secrets of the stability of the HIV-1 capsid. Elife 7:e38895. doi:10.7554/eLife.38895. 30063007 PMC6067877

[60] Dick RA, Zadrozny KK, Xu C, Schur FKM, Lyddon TD, Ricana CL, Wagner JM, Perilla JR, Ganser-Pornillos BK, Johnson MC, Pornillos O, Vogt VM 2018 Inositol phosphates are assembly co-factors for HIV-1. Nature 560:509–512. doi:10.1038/s41586-018-0396-4. 30069050 PMC6242333

[61] Dick RA, Mallery DL, Vogt VM, James LC 2018 IP6 regulation of HIV capsid assembly, stability, and uncoating. Viruses 10:E640. 30445742 10.3390/v10110640 PMC6267275

[62] Winn MD, Ballard CC, Cowtan KD, Dodson EJ, Emsley P, Evans PR, Keegan RM, Krissinel EB, Leslie AG, McCoy A, McNicholas SJ, Murshudov GN, Pannu NS, Potterton EA, Powell HR, Read RJ, Vagin A, Wilson KS 2011 Overview of the CCP4 suite and current developments. Acta Crystallogr D Biol Crystallogr 67:235–242. doi:10.1107/S0907444910045749. 21460441 PMC3069738

[63] Lu M, Hou G, Zhang H, Suiter CL, Ahn J, Byeon IJ, Perilla JR, Langmead CJ, Hung I, Gor'kov PL, Gan Z, Brey W, Aiken C, Zhang P, Schulten K, Gronenborn AM, Polenova T 2015 Dynamic allostery governs cyclophilin A-HIV capsid interplay. Proc Natl Acad Sci U S A 112:14617–14622. doi:10.1073/pnas.1516920112. 26553990 PMC4664340

[64] Liu C, Perilla JR, Ning J, Lu M, Hou G, Ramalho R, Himes BA, Zhao G, Bedwell GJ, Byeon IJ, Ahn J, Gronenborn AM, Prevelige PE, Rousso I, Aiken C, Polenova T, Schulten K, Zhang P 2016 Cyclophilin A stabilizes the HIV-1 capsid through a novel non-canonical binding site. Nat Commun 7:10714. doi:10.1038/ncomms10714. 26940118 PMC4785225

[65] Noviello CM, Lopez CS, Kukull B, McNett H, Still A, Eccles J, Sloan R, Barklis E 2011 Second-site compensatory mutations of HIV-1 capsid mutations. J Virol 85:4730–4738. doi:10.1128/JVI.00099-11. 21367891 PMC3126181

[66] Byeon IJ, Meng X, Jung J, Zhao G, Yang R, Ahn J, Shi J, Concel J, Aiken C, Zhang P, Gronenborn AM 2009 Structural convergence between Cryo-EM and NMR reveals intersubunit interactions critical for HIV-1 capsid function. Cell 139:780–790. doi:10.1016/j.cell.2009.10.010. 19914170 PMC2782912

[67] Tsiang M, Niedziela-Majka A, Hung M, Jin D, Hu E, Yant S, Samuel D, Liu X, Sakowicz R 2012 A trimer of dimers is the basic building block for human immunodeficiency virus-1 capsid assembly. Biochemistry 51:4416–4428. doi:10.1021/bi300052h. 22564075

[68] Ravindranath PA, Sanner MF 2016 AutoSite: an automated approach for pseudo-ligands prediction-from ligand-binding sites identification to predicting key ligand atoms. Bioinformatics 32:3142–3149. doi:10.1093/bioinformatics/btw367. 27354702 PMC5048065

[69] Webb B, Sali A 2014 Comparative protein structure modeling using MODELLER. Curr Protoc Bioinformatics 47:5.6.1–5.6.32. doi:10.1002/0471250953.bi0506s47. 25199792

[70] Pronk S, Pall S, Schulz R, Larsson P, Bjelkmar P, Apostolov R, Shirts MR, Smith JC, Kasson PM, van der Spoel D, Hess B, Lindahl E 2013 GROMACS 4.5: a high-throughput and highly parallel open source molecular simulation toolkit. Bioinformatics 29:845–854. doi:10.1093/bioinformatics/btt055. 23407358 PMC3605599

[71] Hornak V, Abel R, Okur A, Strockbine B, Roitberg A, Simmerling C 2006 Comparison of multiple Amber force fields and development of improved protein backbone parameters. Proteins 65:712–725. doi:10.1002/prot.21123. 16981200 PMC4805110

[72] Mahoney MW, Jorgensen WL 2000 A five-site model for liquid water and the reproduction of the density anomaly by rigid, nonpolarizable potential functions. J Chem Phys 112:8910–8922. doi:10.1063/1.481505.

[73] Berendsen HJC, Postma JPM, van Gunsteren WF, DiNola A, Haak JR 1984 Molecular-dynamics with coupling to an external bath. J Chem Phys 81:3684–3690. doi:10.1063/1.448118.

[74] Parrinello M, Rahman A 1981 Polymorphic transitions in single-crystals - a new molecular-dynamics method. J Appl Phys 52:7182–7190. doi:10.1063/1.328693.

[75] Hess B, Bekker H, Berendsen HJC, Fraaije JGEM 1997 LINCS: a linear constraint solver for molecular simulations. J Comput Chem 18:1463–1472. doi:10.1002/(SICI)1096-987X(199709)18:12<1463::AID-JCC4>3.0.CO;2-H.

[76] Darden T, Perera L, Li L, Pedersen L 1999 New tricks for modelers from the crystallography toolkit: the particle mesh Ewald algorithm and its use in nucleic acid simulations. Structure 7:R55–R60. doi:10.1016/S0969-2126(99)80033-1. 10368306

[77] Craveur P, Joseph AP, Esque J, Narwani TJ, Noel F, Shinada N, Goguet M, Leonard S, Poulain P, Bertrand O, Faure G, Rebehmed J, Ghozlane A, Swapna LS, Bhaskara RM, Barnoud J, Teletchea S, Jallu V, Cerny J, Schneider B, Etchebest C, Srinivasan N, Gelly JC, de Brevern AG 2015 Protein flexibility in the light of structural alphabets. Front Mol Biosci 2:20. doi:10.3389/fmolb.2015.00020. 26075209 PMC4445325

[78] de Brevern AG, Etchebest C, Hazout S 2000 Bayesian probabilistic approach for predicting backbone structures in terms of protein blocks. Proteins 41:271–287. doi:10.1002/1097-0134(20001115)41:3<271::AID-PROT10>3.0.CO;2-Z. 11025540

[79] Joseph AP, Agarwal G, Mahajan S, Gelly JC, Swapna LS, Offmann B, Cadet F, Bornot A, Tyagi M, Valadie H, Schneider B, Etchebest C, Srinivasan N, De Brevern AG 2010 A short survey on protein blocks. Biophys Rev 2:137–147. doi:10.1007/s12551-010-0036-1. 21731588 PMC3124139

[80] Barnoud J, Santuz H, Craveur P, Joseph AP, Jallu V, de Brevern AG, Poulain P 2017 PBxplore: a tool to analyze local protein structure and deformability with Protein Blocks. PeerJ 5:e4013. doi:10.7717/peerj.4013. 29177113 PMC5700758

[81] Wodrich H, Schambach A, Krausslich HG 2000 Multiple copies of the Mason-Pfizer monkey virus constitutive RNA transport element lead to enhanced HIV-1 Gag expression in a context-dependent manner. Nucleic Acids Res 28:901–910. doi:10.1093/nar/28.4.901. 10648781 PMC102582

[82] Longo PA, Kavran JM, Kim MS, Leahy DJ 2013 Transient mammalian cell transfection with polyethylenimine (PEI). Methods Enzymol 529:227–240. doi:10.1016/B978-0-12-418687-3.00018-5. 24011049 PMC4012321

[83] Lanman J, Sexton J, Sakalian M, Prevelige PE Jr. 2002 Kinetic analysis of the role of intersubunit interactions in human immunodeficiency virus type 1 capsid protein assembly in vitro. J Virol 76:6900–6908. doi:10.1128/JVI.76.14.6900-6908.2002. 12072491 PMC136311

[84] Kabsch W 2010 Xds. Acta Crystallogr D Biol Crystallogr 66:125–132. doi:10.1107/S0907444909047337. 20124692 PMC2815665

[85] Evans P 2006 Scaling and assessment of data quality. Acta Crystallogr D Biol Crystallogr 62:72–82. doi:10.1107/S0907444905036693. 16369096

[86] Adams PD, Grosse-Kunstleve RW, Hung LW, Ioerger TR, McCoy AJ, Moriarty NW, Read RJ, Sacchettini JC, Sauter NK, Terwilliger TC 2002 PHENIX: building new software for automated crystallographic structure determination. Acta Crystallogr D Biol Crystallogr 58:1948–1954. doi:10.1107/S0907444902016657. 12393927

[87] Emsley P, Cowtan K 2004 Coot: model-building tools for molecular graphics. Acta Crystallogr D Biol Crystallogr 60:2126–2132. doi:10.1107/S0907444904019158. 15572765

[88] Murshudov GN, Skubak P, Lebedev AA, Pannu NS, Steiner RA, Nicholls RA, Winn MD, Long F, Vagin AA 2011 REFMAC5 for the refinement of macromolecular crystal structures. Acta Crystallogr D Biol Crystallogr 67:355–367. doi:10.1107/S0907444911001314. 21460454 PMC3069751

