# Supplementary figures and images for: Novel Intersubunit Interaction Critical for HIV-1 Core Assembly Defines a Potentially Targetable Inhibitor Binding Pocket
Source: mBio. 2019 Mar 12;10(2):e02858-18. doi: 10.1128/mBio.02858-18 (PMC6414707; doi:10.1128/mBio.02858-18)

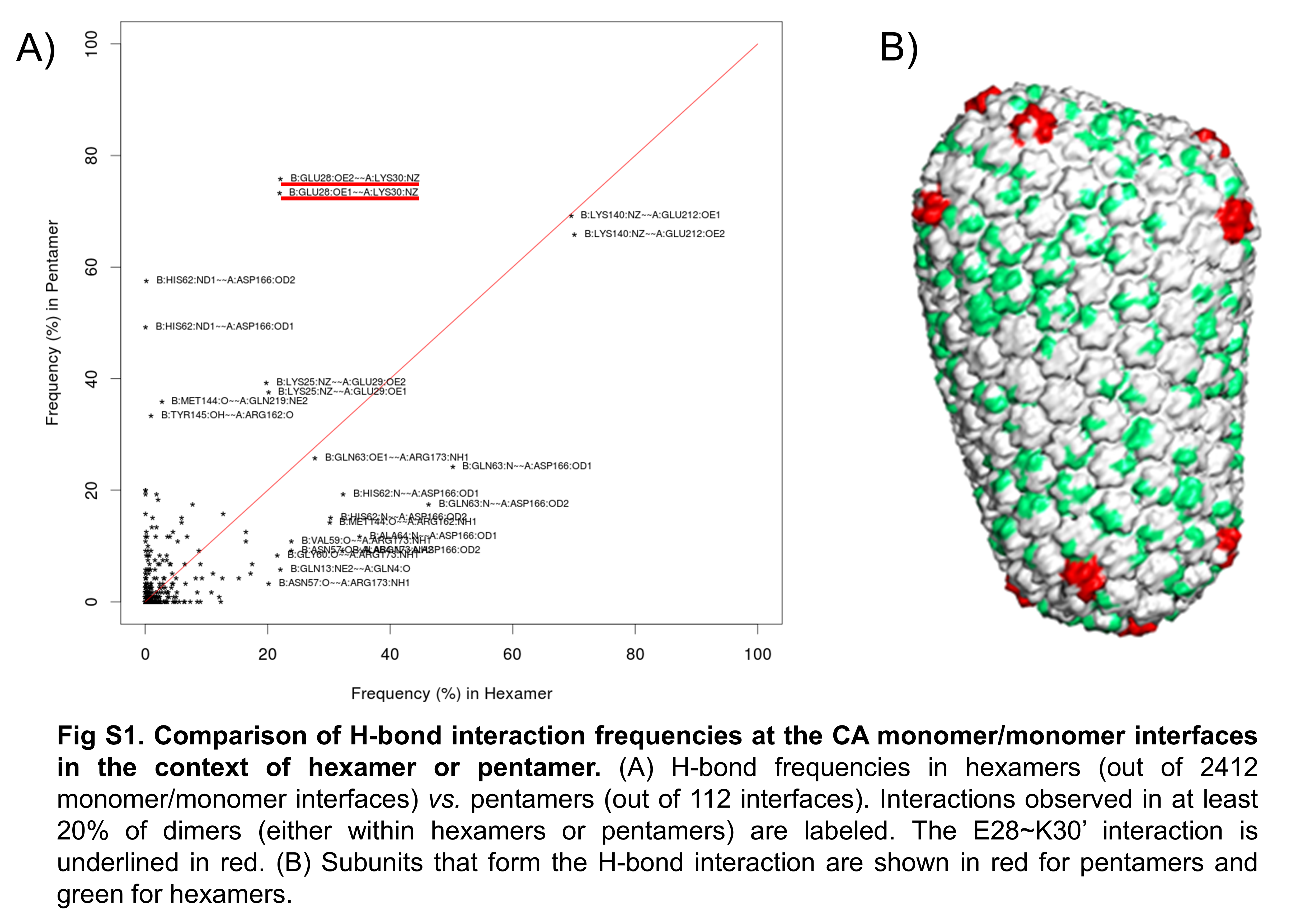

Supplement: FIG S1 [file mBio.02858-18-sf001.tif]

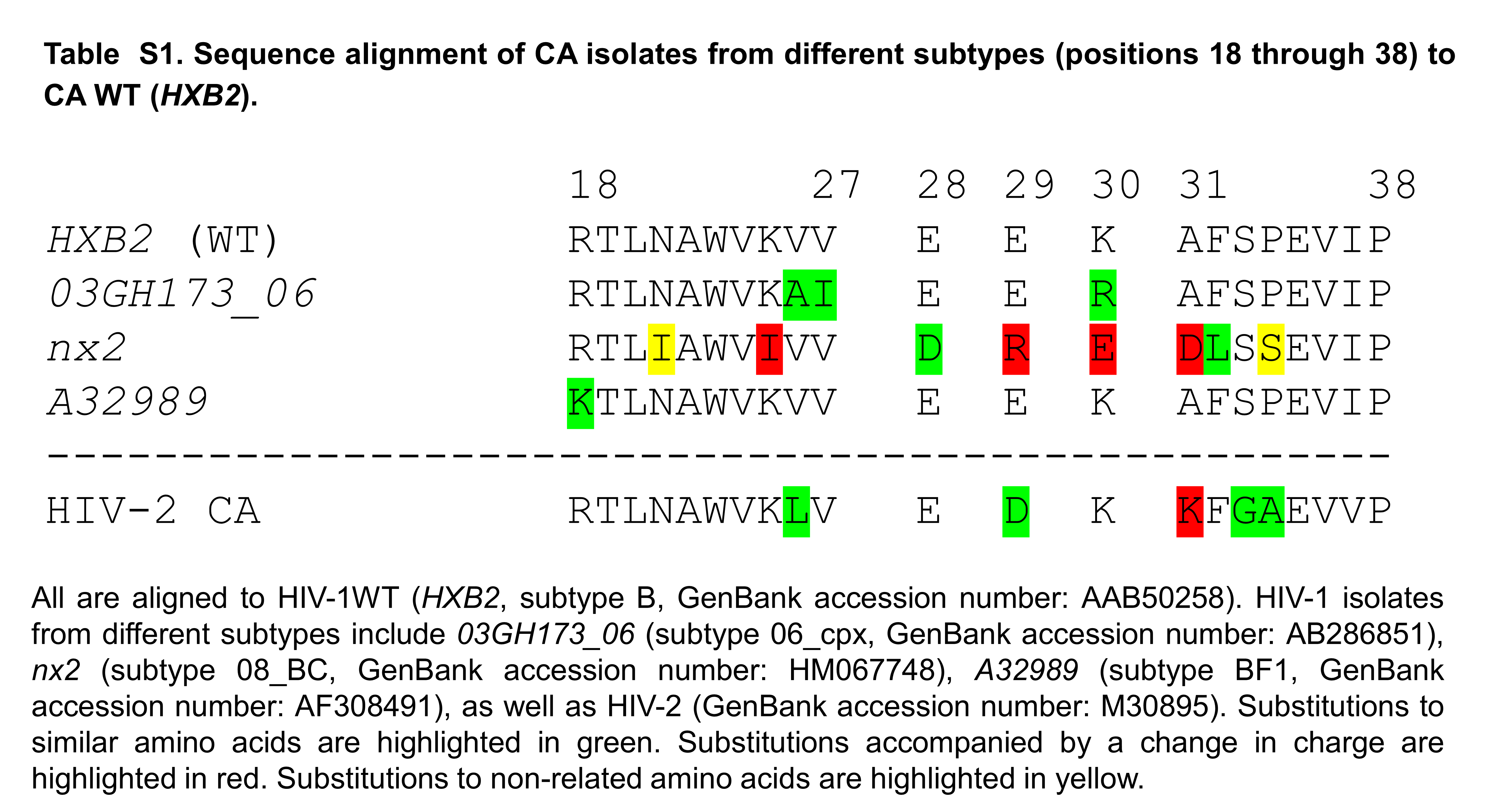

Supplement: TABLE S1 [file mBio.02858-18-st001.tif]

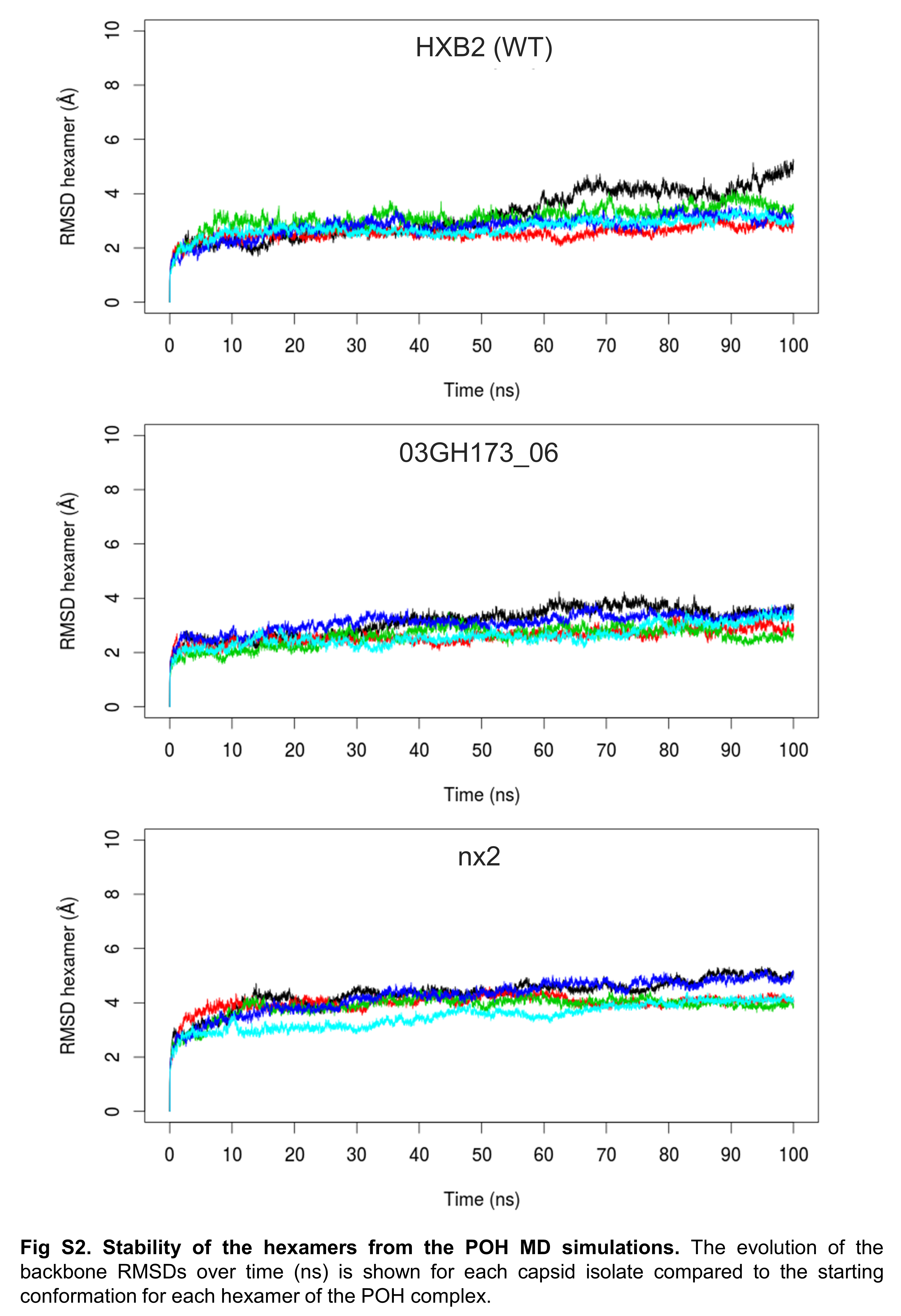

Supplement: FIG S2 [file mBio.02858-18-sf002.tif]

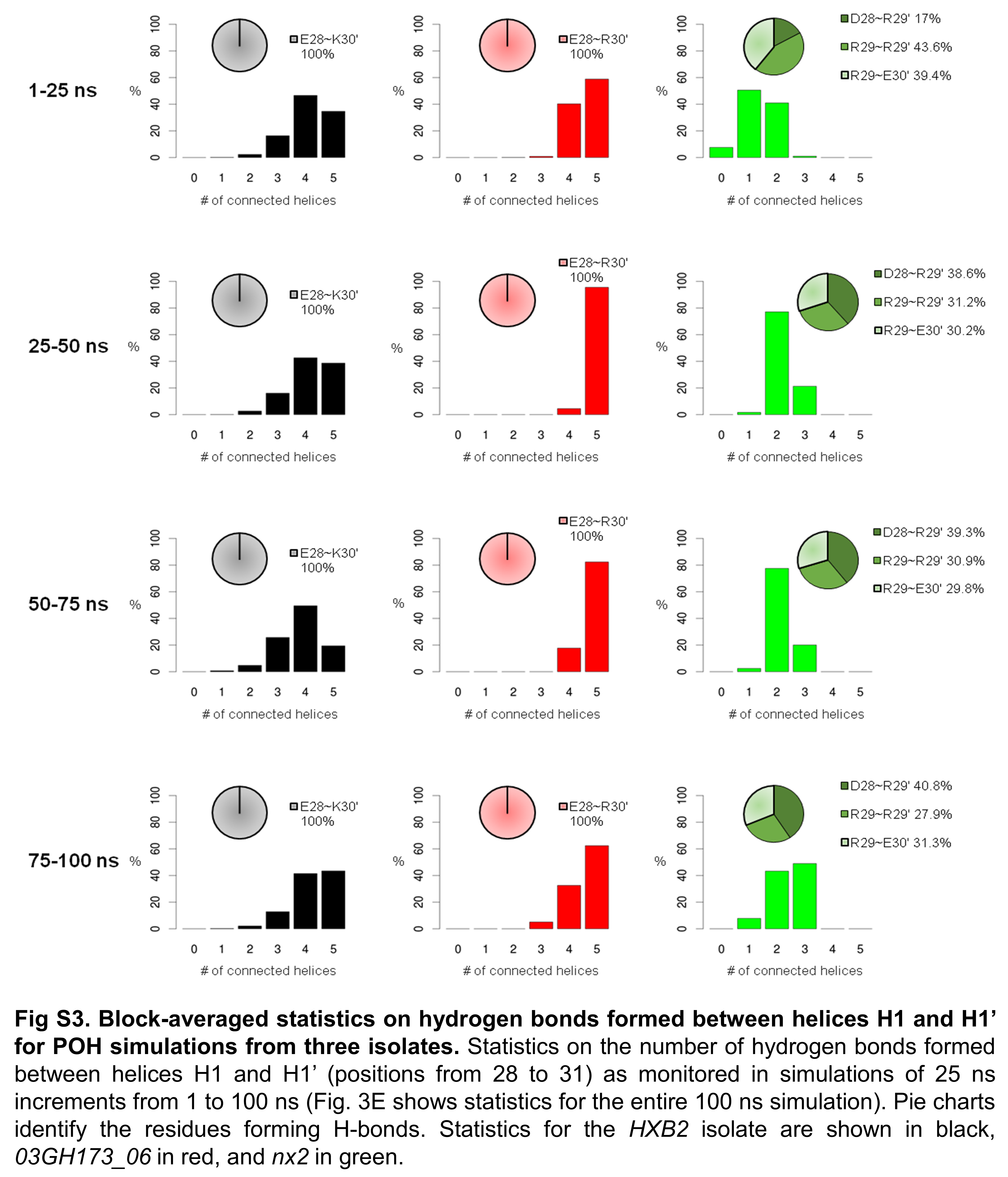

Supplement: FIG S3 [file mBio.02858-18-sf003.tif]

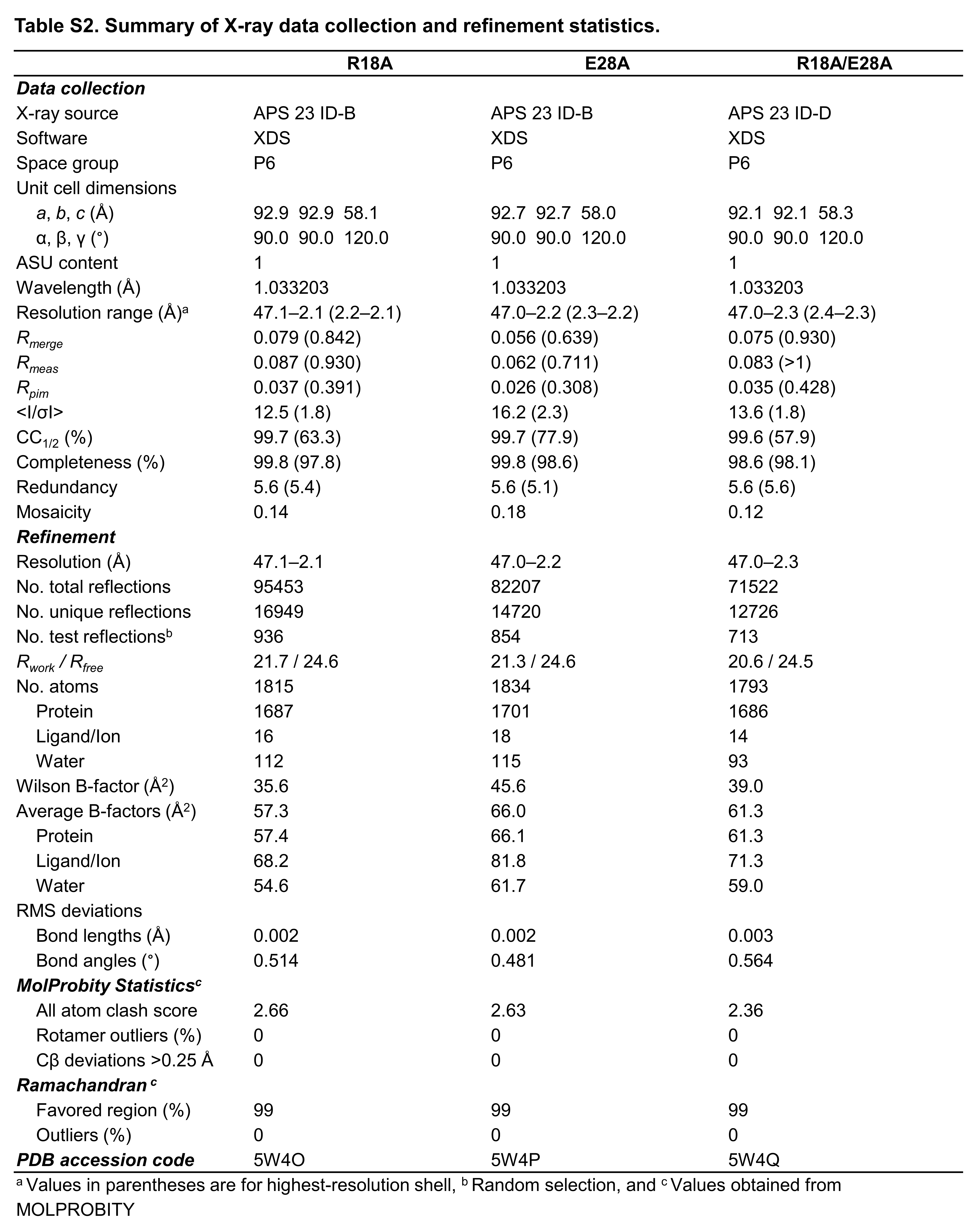

Supplement: TABLE S2 [file mBio.02858-18-st002.tif]

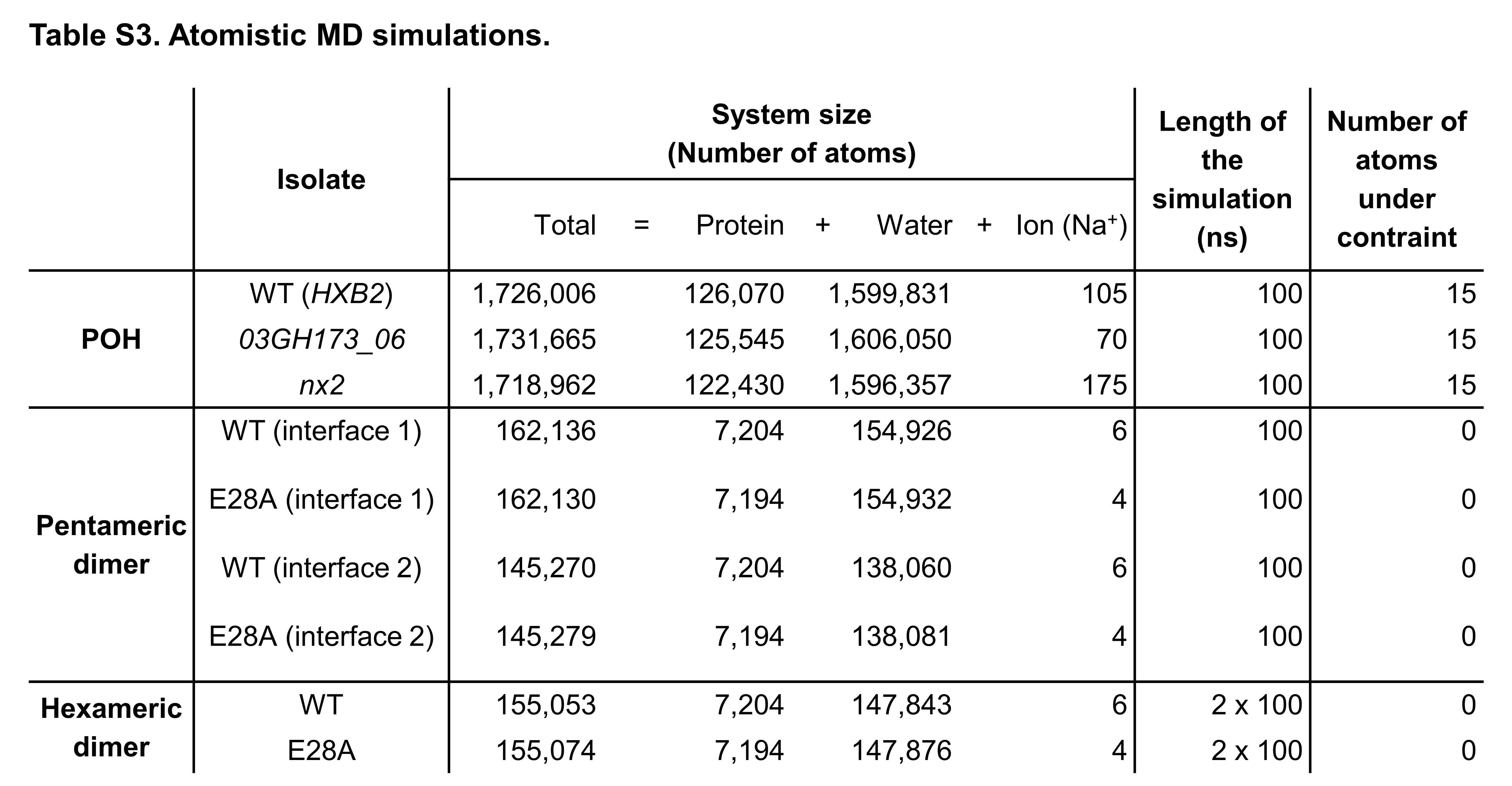

Supplement: TABLE S3 [file mBio.02858-18-st003.tif]

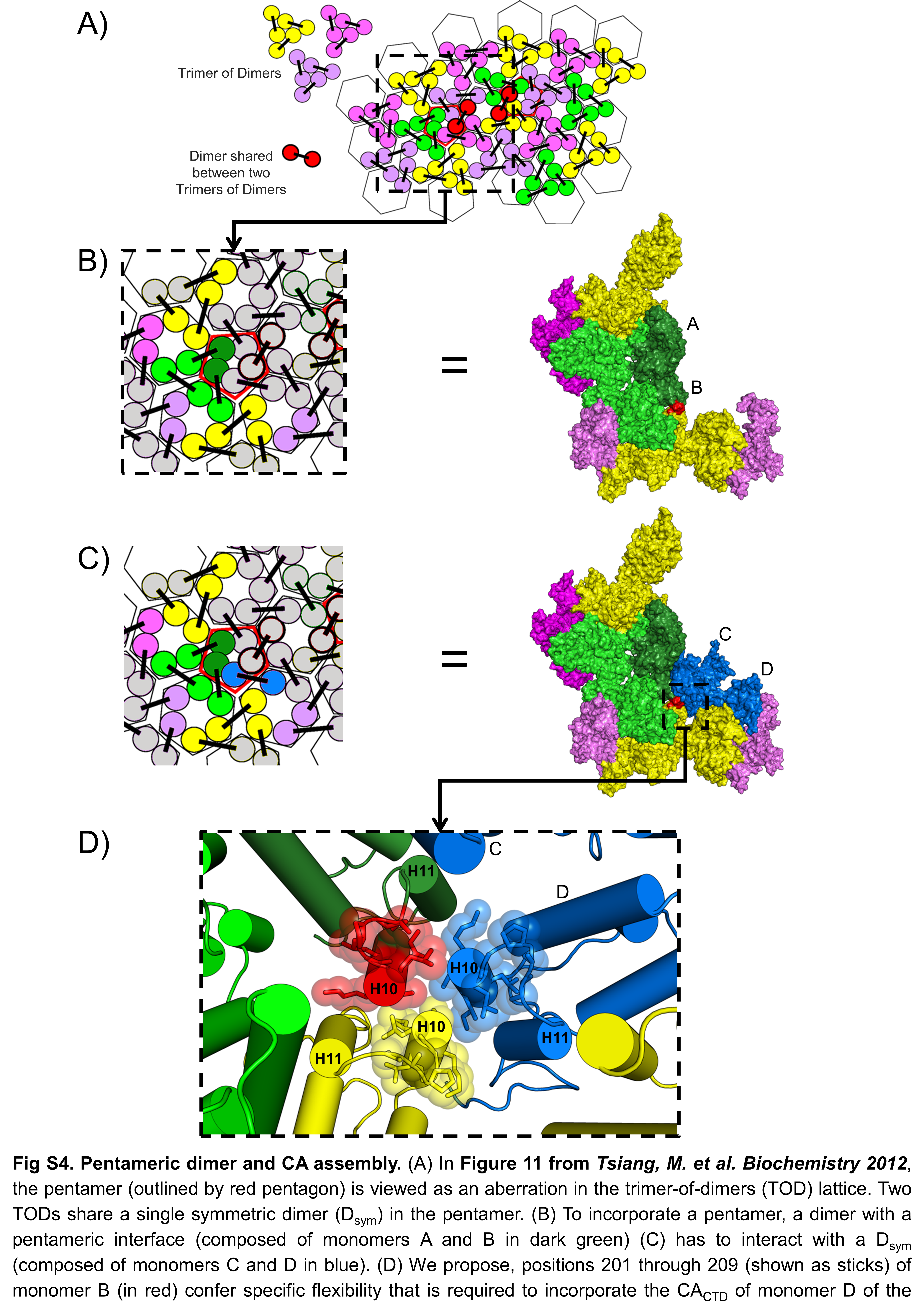

Supplement: FIG S4 [file mBio.02858-18-sf004.tif]
